# Unravelling marker trait associations linking nutritional value with pigmentation in rice seed

**DOI:** 10.1002/tpg2.20360

**Published:** 2023-08-17

**Authors:** Edwige Gaby Nkouaya Mbanjo, Erstelle A. Pasion, Huw Jones, Socorro Carandang, Gopal Misra, John Carlos Ignacio, Tobias Kretzschmar, Nese Sreenivasulu, Lesley Ann Boyd

**Affiliations:** ^1^ International Rice Research Institute (IRRI) Los Baños Philippines; ^2^ International Institute of Tropical Agriculture (IITA) Ibadan Nigeria; ^3^ National Institute of Agricultural Botany (NIAB) Cambridge UK; ^4^ King Abdullah University of Science and Technology Thuwal Saudi Arabia; ^5^ Faculty of Science and Engineering Southern Cross University East Lismore New South Wales Australia

## Abstract

While considerable breeding effort has focused on increasing the yields of staple crops such as rice and the levels of micronutrients such as iron and zinc, breeding to address the problems of the double‐burden of malnutrition has received less attention. Pigmented rice has higher nutritional value and greater health benefits compared to white rice. However, the genetic associations underlying pericarp coloration and accumulation of nutritionally valuable compounds is still poorly understood. Here we report the targeted genetic analysis of 364 rice accessions, assessing the genetic relationship between pericarp coloration (measured using multi‐spectral imaging) and a range of phenolic compounds with potential nutritional and health‐promoting characteristics. A genome‐wide association study resulted in the identification of over 280 single nucleotide polymorphisms (SNPs) associated with the traits of interest. Many of the SNPs were associated with more than one trait, colocalization occurring between nutritional traits, and nutritional and color‐related traits. Targeted association analysis identified 67 SNPs, located within 52 candidate genes and associated with 24 traits. Six haplotypes identified within the genes *Rc/bHLH17* and *OsIPT5* indicated that these genes have an important role in the regulation of a wide range of phenolic compounds, and not only those directly conferring pericarp color. These identified genetic linkages between nutritionally valuable phenolic compounds and pericarp color present not only a valuable resource for the enhancement of the nutritional value of rice but an easy method of selection of suitable genotypes.

## Core Idea

- *Rc/bHLH17* and *OsIPT5* are associated with a range of nutritionally valuable compounds.
- Additional rice candidate genes associated with nutritional value were identified.
- Linkages between color‐related phenotypes and nutritional content were found.

**Abbreviations**
AMOVA: analysis of molecular variance
bHLH: basic helix‐loop‐helix
Bp: base pairs
CV: coefficient of variation
FIS: inbreeding coefficient
GBS: genotyping by sequencing
GI: glycemic index
GLM: generalized linear model
GWAS: genome‐wide association study (GWAS
He: expected heterozygosity
Ho: observed heterozygosity
HPLC: high‐performance liquid chromatography
LD: linkage disequilibrium
MAF: minor allele frequency
MLM: mixed linear model
MTA: marker trait association
Na: number of alleles
PCA: principal component analysis
Q‐Q: quantile‐quantile
QTL: quantitative trait loci
RIL: recombinant inbred line
SD: standard deviation
SNP: single nucleotide polymorphism

## 1 INTRODUCTION

As the world's population continues to grow, being projected to reach 10.4 billion by 2100 (UN DESA PD, 2022), crop productivity needs to be increased to meet the associated demand for food. However, increasing yields alone does not address the increasing global problems of the double burden of malnutrition. This is particularly relevant for the staple crop rice, which is mostly consumed as polished, white rice. The milling process removes many essential nutritional components, including essential micronutrients such as iron and zinc, vitamins, fatty‐acids, phytochemicals, and fiber. This can lead to the development of micronutrient deficiencies among peoples for whom rice is the primary source of calories (Sarma et al., 2018; Sharma et al., 2013; Verma & Shukla, 2011). Furthermore, white rice tends to have a high glycemic index (GI), the starch being rapidly digested in the human intestine (Oko et al., 2012; Valarmathi et al., 2014). The consumption of high GI and glycemic load foods, alongside a decrease in physical activity and rapid urbanization, has contributed to the expansion of non‐communicable diseases such as type 2 diabetes, with the Philippines having the highest recorded levels (Ludwig et al., 2018; Saneei et al., 2016). Thus, high consumption of white rice presents as a prime example of the double burden of malnutrition.

Variation in pericarp color is well known in rice, with colors ranging from brown, red, purple to black. Prized in ancient times, pigmented rice is regaining popularity owing to its higher nutritional value (Mbanjo et al., 2020; Priya et al., 2019). The deposition of flavonoid and anthocyanin compounds in the seed pericarp layer, responsible for the color of unpolished rice (Gunaratne et al., 2013; Samyor et al., 2017), also confer many positive health benefits, having anti‐cancer, anti‐diabetic, and anti‐hyperlipidemic properties (Berni et al., 2018; Bhat & Riar, 2015). While major enzymes of the flavonoid pathways have been identified, many aspects underlying the regulation of these pathways in rice seed are less well understood (Mbanjo et al., 2020).

The ancestor of modern rice varieties had red grain. The wild rice species *Oryza rufipogon* has red grains and is thought to have a common ancestor with *Oryza sativa*. The intensity of the red coloration is determined by a complementary interaction between the gene *Rc* and a second gene, *Rd* (Furukawa et al., 2006; Sweeney et al., 2006). While *Rc* is responsible for the accumulation of proanthocyanidins in the pericarp, *Rd* regulates the level of accumulation. A loss of function mutation within the *Rc* gene (a 14–bp deletion within exon 6) is thought to be the original mutation that gave rise to the white rice *rc* allele (Furukawa et al., 2006; Sweeney et al., 2006). Singh et al. (2017) identified an independent haplotype, *Rc‐H2*, which was associated with white pericarps in the Aus group of rice cultivars. Subsequent studies have identified additional mutations within the *Rc* gene, including the *Rc‐S*, *rc‐gl*, and *Rc‐g* alleles that result in reversion to red pigmentation (Brooks et al., 2008; Gross et al., 2010; Singh et al., 2017; Sweeney et al., 2007). More recently, Mbanjo et al. (2019) identified other possible mutations, either in *Rc* or other genes, that affect pericarp coloration.

**Core Idea**

- *Rc/bHLH17* and *OsIPT5* are associated with a range of nutritionally valuable compounds.
- Additional rice candidate genes associated with nutritional value were identified.
- Linkages between color‐related phenotypes and nutritional content were found.

The regulation of anthocyanins, responsible for black‐purple rice pericarps, is less well understood. Early studies identified two classes of regulatory gene families (*R* or *B*, and *C1* or *Pl*) that affect anthocyanin accumulation and regulate the deposition of anthocyanin in various tissues in rice. Two genes were characterized in rice, *Ra* and *Rb*, which mapped to chromosomes 4 and 1, respectively (Hu et al., 1996). Maeda et al. (2014) identified three loci that controlled black pigmentation in the rice pericarp, *Kala1*, *Kala3*, and *Kala4* on chromosomes 1, 3, and 4, respectively. Ectopic expression of *Kala4*, a member of the basic helix‐loop‐helix (bHLH) transcription factor gene family, caused by a rearrangement in the promoter region, was shown to result in black seeded rice (Oikawa et al., 2015). The C‐S‐A gene model has also been proposed as a model for rice seed coloration, the pattern of anthocyanin pigmentation being determined by the allelic status of the genes *A1*, *C1*, and *S1* (Rachasima et al., 2017; Saitoh et al., 2004; X. Sun et al., 2018). The presence of a functional copy of both *C1* and *S1* is required for pericarp coloration, while *A1* acts as a catalyst for the development of purple pericarps (X. Sun et al., 2018). Sequence variants in other regulatory and/or structural genes associated with purple coloration could also explain the diversity of phenotypes observed (Rachasima et al., 2017; Sakulsingharoj et al., 2016; X. Sun et al., 2018).

Interrogation of the genetic basis underlying rice pericarp color has also identified a number of quantitative trait loci (QTL; Dong et al., 2008; Matsuda et al., 2012; Tan et al., 2001; Xu et al., 2017). Tan et al. (2001) identified nine QTL in an analysis of flour color parameters using 238 recombinant inbred lines (RILs), while Dong et al. (2008), measuring the extent of the red coloration of the pericarp, found four QTL in a population of 182 RILs. In Dong et al. (2008), the two largest QTL co‐located with the genetic loci *Rc* on chromosome 7 and *Rd* on chromosome 1. A QTL analysis using 85 lines suggested that flavonoid content was governed by genetic factors that controlled flavone glycosylation (Matsuda et al., 2012). More recently, 21 QTL were identified that affected the composition and concentration of anthocyanins and proanthocyanidins (Xu et al., 2017). Using a diversity panel of 416 rice accessions, Shao et al. (2011) identified 25 marker‐trait associations (MTAs) with color‐related traits, confirming the major role of *Ra* and *Rc*, but also identifying a number of genetic regions with minor effects. Yang et al. (2018) reported 763 single nucleotide polymorphisms (SNPs) tightly linked to pericarp color in rice landrace germplasm, further confirming the polygenic nature of rice pericarp coloration and demonstrating the need to capture this genetic variation for rice breeding.

Developments in genetic technologies now offers new prospects for crop improvement. Low‐cost and parallel sequencing platforms such as genotyping‐by‐sequencing (GBS) have facilitated high‐density marker discovery for crop genetic studies, while cutting‐edge analytical technologies have enabled sensitive and accurate detection of the genetic loci associated with the trait of interest (Elshire et al., 2011; He et al., 2014; Mgonja et al., 2017). Spectral imaging techniques have been increasingly used as a rapid phenotyping tool to quantify several seed traits (Elmasry et al., 2019; Mbanjo et al., 2019; D. Sun et al., 2019) and enable efficient assessment of the phenotypic diversity of rice germplasm. Together, the improvements in phenotypic and genotypic analyses support the extensive mining of natural variation in rice germplasm, and discovery of new genes/alleles associated with important rice traits such as those associated with grain coloration.

The aim of this study was to identify genetic associations between pericarp color parameters and nutritional traits that would enable a better understanding of the genetic regulation of these characteristics, while providing simple seed phenotypes that could be screened for by breeders to improve nutritional value in rice. To this aim, we assessed a range of grain nutritional and color parameters, using multispectral imaging, in 364 rice accessions from the International Rice Research Institute (IRRI) Genebank. The rice accessions were subjected to genotyping‐by‐sequencing, enabling high‐density genome‐wide SNP markers to be generated. The SNP markers were used to estimate linkage disequilibrium (LD) decay, assess genetic diversity and population structure within the rice germplasm, and perform genome‐wide association mapping using the seed traits. Candidate genes within genomic regions identified as showing a highly significant MTA were further analyzed via targeted gene‐based association and haplotype analyses.

## 2 MATERIALS AND METHODS

### 2.1 Plant material

A total of 376 rice accessions from 24 countries were selected from the IRRI Genebank (Table S1). Accessions were chosen to generate a panel of rice genotypes that presented a full range of colored grain, represented the geographical diversity across South‐East Asia, and included both traditional rice varieties and landraces. Visual assessment of color was undertaken using Biodiversity indicators for pericarp color (Bioversity International et al., 2007).

### 2.2 DNA extraction and genotyping‐by‐sequencing

Fresh leaf material from seedlings of each rice accession was lyophililized using an Alpha 1–4LD plus Martin Christ Freeze dryer for 72 h, with vacuum set at 0.630 mbar and condenser temperature at −55˚C. DNA extraction was performed using the KingFisher Flex System (www.thermofisher.com). DNA quality was checked on 1% agarose gels, while DNA quantity was assessed using PicoGreen® (www.thermofisher.com) and then standardize to 100 ng/μL for library preparation. To ensure that the DNA was of high quality and suitable for genotyping‐by‐sequencing (GBS), a trial digest of DNA from 10% of the accessions was performed using the restriction enzyme *ApekI*. The DNA (minimum concentration of 100 ng/μL) was shipped to Elshire Group Ltd (https://www.elshiregroup.co.nz) for GBS library construction and sequencing. Libraries were prepared in 188‐plex using the *ApekI* restriction enzyme, following the standard procedure proposed by Elshire et al. (2011). Paired‐end sequencing of 188‐plex libraries per flow cell channel was performed on the Illumina Hiseq X Ten sequencing platform.

### 2.3 SNP identification

The Fastq raw files received from Elshire Group Ltd. were processed using the workflow proposed by Elshire (https://gitlab.com/relshire/gbs‐pipeline‐scripts) with some modifications. The paired‐end reads were demultiplexed into individual samples using axe‐demux (Murray & Borevitz, 2018). Seqhax (https://github.com/kdmurray91/seqhax) was used to add fake barcodes and append 130 Ns. Seqhax was also used to trim the reads to 130 bp for efficient downstream processing. SNP calling was performed with TASSEL v.5.0 (Bradbury et al., 2007) using the modified enzyme definition. SNP calling was achieved by aligning the tags to the Nipponbare RefSeq genome (Nipponbare IRGSP 1.0 genome). Indels and multiallelic markers were discarded, with only SNP markers being retained. The SNP data were extracted in hapmap file format.

Control quality of the genotypic data was performed using SNP & Variation Suite (SVS v8.8.3) (Golden Helix, Inc., Bozeman, MT, USA). Markers with a call rate (CR) of <0.90 and a minor allele frequency (MAF) of <0.05 were removed from the data set. The observed SNP heterozygosity (Ho)/expected SNP heterozygosity (He) values were calculated for individual SNPs using the hardy function in PLINK 1.9 (Chang et al., 2015). SNP inbreeding coefficients were estimated as *F* = 1 − Ho/He. A cut‐off Ho/He <0.5 was used to remove SNPs with an excessive number of heterozygotes. The remaining markers were pruned using SNP & Variation Suite (SVS v8.8.3) (Golden Helix) and the default parameters (window size = 50, window increment = 5, *r* ^2^ = 0.5) to produce a set of 5181 markers for LD, population structure, and genetic diversity studies. Rice accessions with a heterozygosity rate above 10% were discarded, leaving 364 accessions.

### 2.4 Linkage disequilibrium

Genome‐wide and chromosome‐specific LD values were estimated based on adjacent and pairwise measurements in SNP & Variation Suite (SVS v8.8.3) (Golden Helix, Inc.) and TASSEL v.5.0 (Bradbury et al., 2007). The genome‐wide LD decay was plotted as LD (*r* ^2^) versus physical distance using the ggplot 2 function (Wickham, 2016) of R statistical software (Rstudio Team, 2016). A cut‐off value of *r* ^2^ = 0.2 was set to estimate the average LD. EM algorithm was used as an interactive technique for obtaining maximum likelihood estimates of sample haplotype frequency.

### 2.5 Population structure

The population structure of the rice accessions was assessed using the selected 5181 SNP markers that reduced the influence of strong LD. The maximum likelihood estimate, implemented in the admixture program (Alexander & Lange, 2011), was used to assign rice accessions to hypothetical groups without prior knowledge of their population affinity. This analysis was carried out using the CoARE facility of the DOST‐Advanced Science and Technology Institute (DOST‐ASTI) and the Computing and Archiving Research Environment (CoARE) project. A 10‐fold cross‐validation procedure was used to estimate the most suitable *K*‐value. The number of *K*‐value computes ranged from 1 to 15. Cross‐validation error estimates were compared to identify the *K*‐value with the lowest cross‐validation error. The proportion of the putative ancestral population of each rice accession was defined in the Q‐matrix. Rice accessions were assigned to a group if the probability of their group membership, as determined by ADMIXTURE, was ≥80%. Accessions with less than 80% probability of a single group membership were classified as admix. Population information of previously characterized accessions was used to identify distinct genetic groups.

Population structure was also assessed using principal component analysis (PCA). The genomic additive relationship matrix was computed using the “A.mat” function from the rrBLUP package in the statistical software R (Endelman, 2011), and PCA was performed using the prcomp command in R. The genetic distance between all possible pairs of rice accessions was calculated using the bitwise.dist function implemented in the R package Poppr (Kamvar et al., 2014). A phylogenetic tree was constructed and visualized in the R package—Analyses of phylogenetics and Evolution (ape) (Paradis et al., 2004).

### 2.6 Genetic diversity

The following genetic metrics were estimated: the number of alleles (Na), observed heterozygosity (Ho), expected heterozygosity (He), and the inbreeding coefficient (FIS). Estimates of genetic diversity were performed with the R package diveRsity, using the divBasic function (Keenan et al., 2013). The genetic metrics He and FIS were estimated for each individual rice accession using Golden Helix SNP & Variation Suite (Golden Helix, Inc.). FIS significance was assessed using a 95% confidence interval and 1000 bootstrap replicates. Significant differences in diversity between groups were estimated using Wilcoxon signed rank test in R (RStudio Team, 2016). Analysis of molecular variance (AMOVA) was performed to estimate the variance between populations and/or groups using R package Poppr (Kamvar et al., 2014). Population differentiation was evaluated using pairwise *F*_ST_ estimate based on Wright F statistics (Weir & Cockerham, 1984) using the R package diversity (Keenan et al., 2013) and the diffCalc function.

### 2.7 Multi‐spectral imaging of rice pericarps

Dehulled rice seed was used for multi‐spectral phenotyping. All digital images were captured using VideometerLab 4 (https://videometer.com) using the method described by Mbanjo et al. (2019). Color difference metrics measured included a* (green to red shade), b* (blue to yellow shade), *L* (lightness, clarity of the pericarp), intensity, saturation (the saturation of a color describes its degree of purity in relation to neutral gray), and hue (angular specification of the color perceived as red, yellow, blue, or green), as well as providing an estimate of anthocyanin levels. Geometric traits assessed were area, length, width, and roundness (an estimate of how closely the shape of the grain resembles a circle).

### 2.8 Nutritional profiling of rice grain

Eighteen phenolic compounds associated with pericarp pigmentation and nutritional potential (referred to as nutritional traits) were quantified in dehulled seed of the 364 retained rice accessions using high‐performance liquid chromatography (HPLC). These compounds included quercetin, kaempferol, catechin, myricetin, flavone, ferulic acid, vanillic acid, syringic acid, gallic acid, cinnamic acid, p‐coumaric acid, protocatechuic acid, peonidin and peonidin glycoside, delphinin and delphinidin glycoside, and cyanidin and cyanidin glycoside. Dehulled, unpolished seeds of each rice accession were milled using a Glen Creston Mill and a sieve size of 2 mm. Flour samples of approximately 2.5 g were extracted into 50 mL of ethanol‐acetic acid (10% 1 M acetic acid v/v) under reflux conditions for 2 h. Extracts were stored at −20°C until analyzed. The extracts were prepared for chromatography by centrifugation for 2 min at 13,000 rpm (approximately 10,000 g) and then filtered through a 0.2 μm filter. The compounds were separated using the Dionex Ultimate 3000 HPLC system. A 150 mm × 4.6 mm × 5 μm × 100 Å Kinetix C18 column was used. The mobile phase was a mixture of 1% formic acid and neat acetonitile, ran at 0.2 ml/min, generating a gradient of 0.95:0.05 for 2 min, 0.72:0.10 for 18 min, 0.00:0.28 for 28 min, and then held until 45 min. The column effluent was monitored with a photo diode array detector between 200 and 600 nm, with data recorded at 254, 280, 340, and 520 nm. Three technical replications were performed on each grain sample.

### 2.9 Phenotype data analysis

The average values from 2 to 3 readings of 20 grain for each accession were calculated and used in further analyses. Descriptive statistics for each trait (minimum and maximum values, range, median, variance, coefficient of variation [CV], standard deviation [SD]) were obtained using the R package pastecs describe function (Grosjean et al., 2018). Boxplots were constructed to assess variability of the phenotypes and histograms to evaluate trait distributions. Correlations between traits and their level of significance were conducted in the R package Hmisc (Harrell et al., 2020) using the function rcorr. Correlograms were generated using library “corrplot.” A correlation network, to visualize interconnectivity between the traits, was built using the R package qgraphic. Phenotypic data were subjected to PCAs using the R package factorMineR (Lê et al., 2008). The relationship between the 364 rice accessions was evaluated using the grain trait phenotypic data sets. Dissimilarity values were computed using the function dist () implemented in R (RStudio Team, 2016) and used with Ward's minimum variance method for hierarchical clustering. The hierarchical clustering result was visualized using ggtree (Yu et al., 2017).

### 2.10 Genome‐wide association study

The Shapiro–Wilk test was used to check phenotype data for normality. The generalized linear model (GLM) with the appropriate distribution was used for traits that did not follow a normal distribution. Outliers were excluded after computing residuals for each trait. The process was repeated until the model fitted the data. A genome‐wide association study (GWAS) was performed in SNP & Variation Suite (SVS v8.8.3) (Golden Helix, Inc.) using the initial data with normally distributed residuals. The geometric and color‐related data obtained from the videometer, multispectral analyses of rice grain, as well as the phenolic data, were used to identify MTAs. Several approaches were assessed, including the naïve and mixed linear model (MLM) approaches. The naïve approach assumes independence of samples, and although the input data were corrected for population stratification, a high inflation rate was obtained (data not shown). Therefore, MLMs implemented in SNP & Variation Suite (SVS v8.8.3) were used. MLM approaches included the single‐locus method developed by Efficient Mixed‐Model Association eXpedited Model (EMMAX), and the multiple‐locus linear mixed model (MLMM; Segura et al., 2012). With EMMAX, one SNP at a time is tested for associations with the phenotypic data set (Kang et al., 2010). MLMM assumes that multiple loci are associated with the phenotype and involves a stepwise implementation of EMMAX with multiple iterations. Cofactors were selected at each stepwise implementation by choosing the SNP with the smallest *p*‐value and adding that as a cofactor in subsequent, forward including steps. A PCA matrix (first three vectors used as fixed covariates) and a GBLUP matrix were used to correct for population stratification and genetic relatedness, respectively. Ten steps were implemented in the MLMM. The optimal step at Bonferroni correction was considered. To evaluate the extent of quantile‐quantile (Q‐Q)‐plot inflation, Q‐Q‐plots were generated by plotting the negative log10‐transformed observed *p*‐values obtained for each SNP marker association against their expected distribution under the null hypothesis of no genetic association. The *p*‐value for each SNP was subject to Bonferroni multiple comparison adjustment. The Bonferroni corrected *p*‐value [−log10(*P*) = ; *P* = 0.05/N], where *N* is the total number of markers, was used as a threshold *p*‐value. Markers exhibiting a Bonferroni *p*‐value ≤7.80 10^−7^ were considered associated with the trait. In addition, we considered a *p*‐value ≤1 × 10^−5^ (−log10(*P*) ≥ 5) as being suggestive of an SNP/trait association. Manhattan plots of associated SNPs were visualized in GenomeBrowse V1.0 (Golden Helix).

### 2.11 Targeted association analysis and functional annotation of target loci

All putative genes lying within the genomic region of significant SNPs, as identified by single‐locus GWAS, were subjected to targeted association analysis. The candidate genes were annotated based on the Nipponbare Reference Genome (MSU7) using ANNOVAR (Wang et al., 2010). All SNPs located in the 2 kb upstream (to include promoter region), genic coding, and 1 kb downstream (to include 3′UTR) regions of each gene were extracted using PLINK (Purcell et al., 2007) and tested for association with each trait of interest using EMMAX (Kang et al., 2010), similar to the method of Misra et al. (2020). The Bonferroni correction method (Haynes, 2013) was used to control the false positive rate and further filter for significant SNPs for each candidate gene, using the formula: *α*/*m*, where *α* = 0.05 and *m* is the number of SNPs per gene. Results were visualized using Cytoscape (Shannon et al., 2003). Edges were defined based on the beta effect of each SNP to the trait of interest, with blue and red representing positive and negative effects, respectively. Source nodes represented the traits of interest, and the target nodes represented the candidate genes.

## 3 RESULTS

### 3.1 Assessment of rice grain color and nutritional traits

The 364 rice accessions originated from 24 countries, primarily collected from South‐East Asia. The number of accessions by country ranged from 1 to 141 (Figure S1, Table S1). Visual assessment of pericarp color qualified 36.54% as red, 24.45% as variable purple, 10.99% as purple, 26.92% as white, with 1.10% being classified as mixed color (Figure S2). The descriptive statistics of the grain geometric and color‐related traits (Table S2), and the nutritional traits (Table S3), are shown in Table S4. The greatest CV was observed in the nutritional parameters, as compared to pericarp color and grain geometric parameters. The quantitative variation within each trait is displayed in boxplots (Figure S3). The Shapiro–Wilk test indicated departure from a normal distribution (*p* < 0.05) for all traits except grain length and width, which showed no significant deviation from normality (*p* > 0.05) (Figure S4).

**FIGURE 1:**
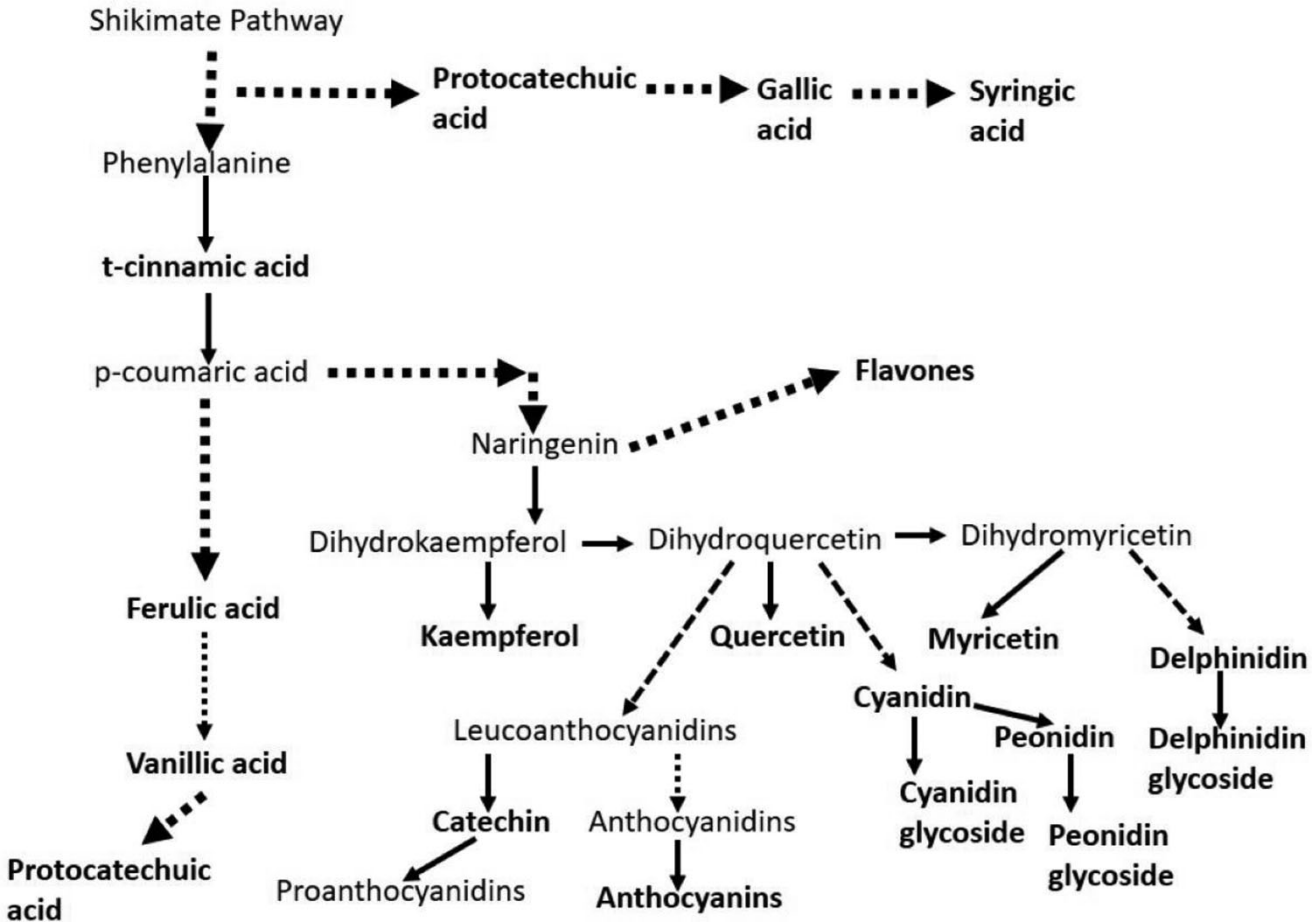
Biochemical pathway of selected phenolic compounds. The schematic shows the biochemical relationship between the phenolic compounds measured in this study (shown in bold) (Katsumoto et al., 2017; Mbanjo et al., 2020).

Analysis of the plant phenolic compounds (Figure 1) revealed differences in whole grain between rice accessions, both within and between color groups (Figure S3). Kaempferol, ferulic acid, and protocatechuic acid were present in all rice accessions, while cyanidin, peonidin, and delphinidin were not detected, being found only in the glycosylated form. Gallic acid was also not detected in any of the rice accessions. Quercetin was detected in 84.6% of the accessions and across all grain color types. Catechin was detected in all accessions, except in one purple accession. Delphinidin glycoside and peonidin glycoside were absent from all of the white rice accessions. Delphinidin glycoside was only found in purple and variable purple rice accessions. Peonidin glycoside was predominately present in purple and variable purple rice accessions but was also found in three red accessions. Delphinidin glycoside and peonidin glycoside are recognized as the dominant anthocyanins in rice varieties with black and purple grain (Frank et al., 2012; Shao et al., 2018; Sompong et al., 2011). Cyanidin glycoside, a precursor of anthocyanin, was more abundant in purple and variable purple rice accessions. Vanillic acid, cinnamic acid, and p‐coumaric acid were detected in all color groups, and in nearly all accessions. Syringic acid was also reported in all the rice grain color groups. However, a large proportion of red rice accessions (64%) and a considerable number of white rice accessions (19.4%) were devoid of syringic acid. Flavone was present in 27.7% of the rice accessions.

**FIGURE 2:**
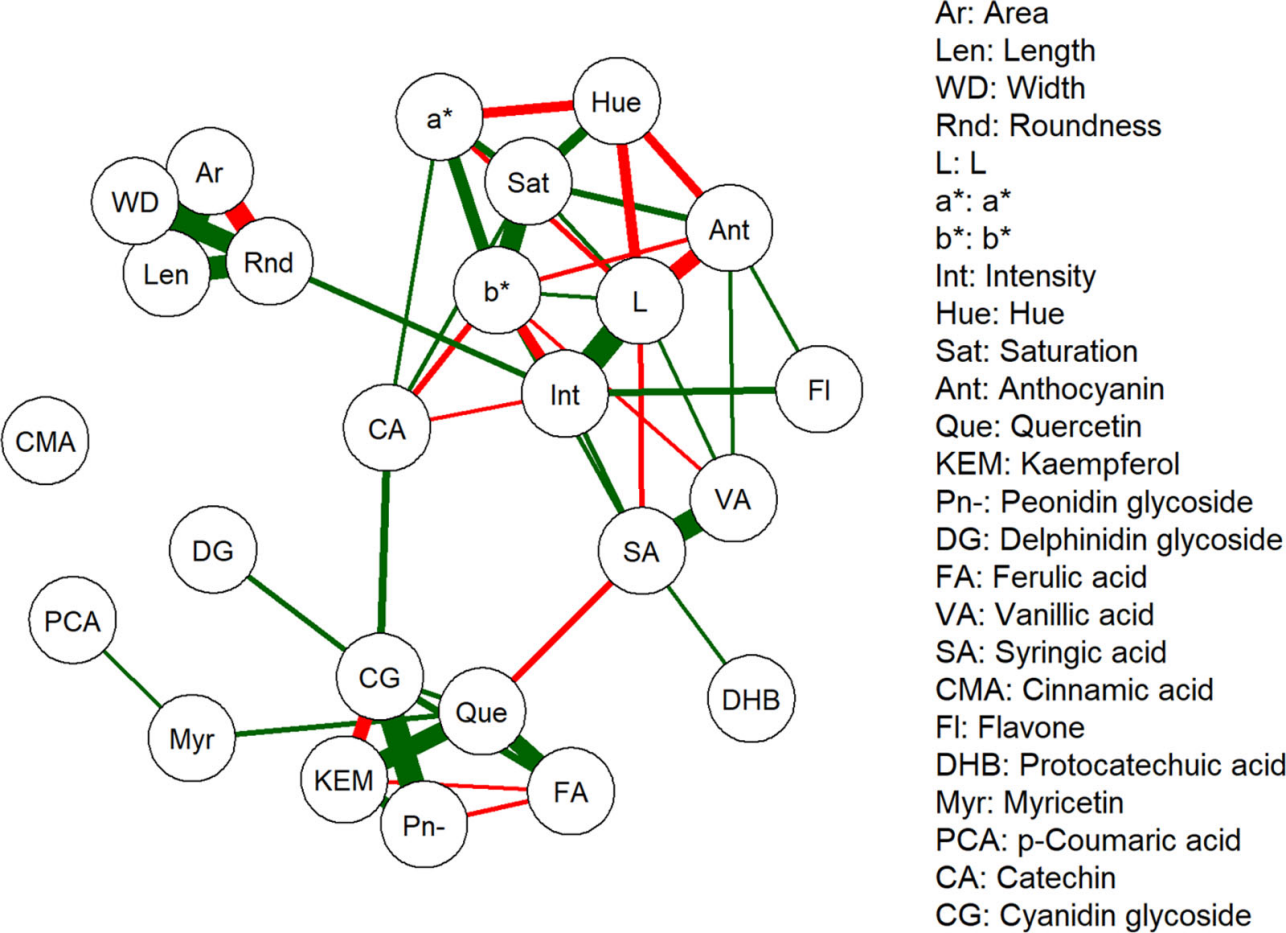
Correlation‐based network visualization of geometric, color‐related and nutritional traits. Correlation was computed using the rcorr function implemented in the R package Hmisc, and the correlation‐based network was constructed using the R package qgraph (Re). Only significant correlations are depicted. The traits are presented as nodes and their relationships as links. Positive correlations are denoted as green edges and negative correlations as red edges. The size of the link corresponds to their relative connectedness.

The interconnectivity between geometric, color‐related and nutritional traits was revealed by the network visualization analysis (Figure 2) and Pearson's correlation (Figure S5). The network visualization analysis (Figure 2) highlighted the interconnectivity between geometric traits, as well as between videometric color traits. Phenolic compounds that are closely associated through their biochemical synthesis pathways (Figure 1) also clustered together. As expected, network 1 consists of quercetin, kaempferol, cyanidin glycoside, and peonidin glycoside; however, ferulic acid was also part of this cluster, despite being on a different part of the shikimate pathway. Network 2 consisted of the color parameters *L*, a*, b*, intensity, saturation and hue, and anthocyanin. Catachin, a flavonoid, connected the two networks. Cinnamic acid was not connected to any of the other traits measured. Similarly, Pearson's correlation analysis indicated significant positive correlations between the color parameters saturation and b*, and *L* and intensity. The highest positive correlation between nutritional parameters was observed between vanillic acid and syringic acid. Anthocyanin levels (measured using the VideometerLAB4) exhibited a strong negative correlation with *L* and intensity, while vanillic and syringic acid exhibited strong negative correlations with *L*, intensity, b*, and saturation.

**FIGURE 3:**
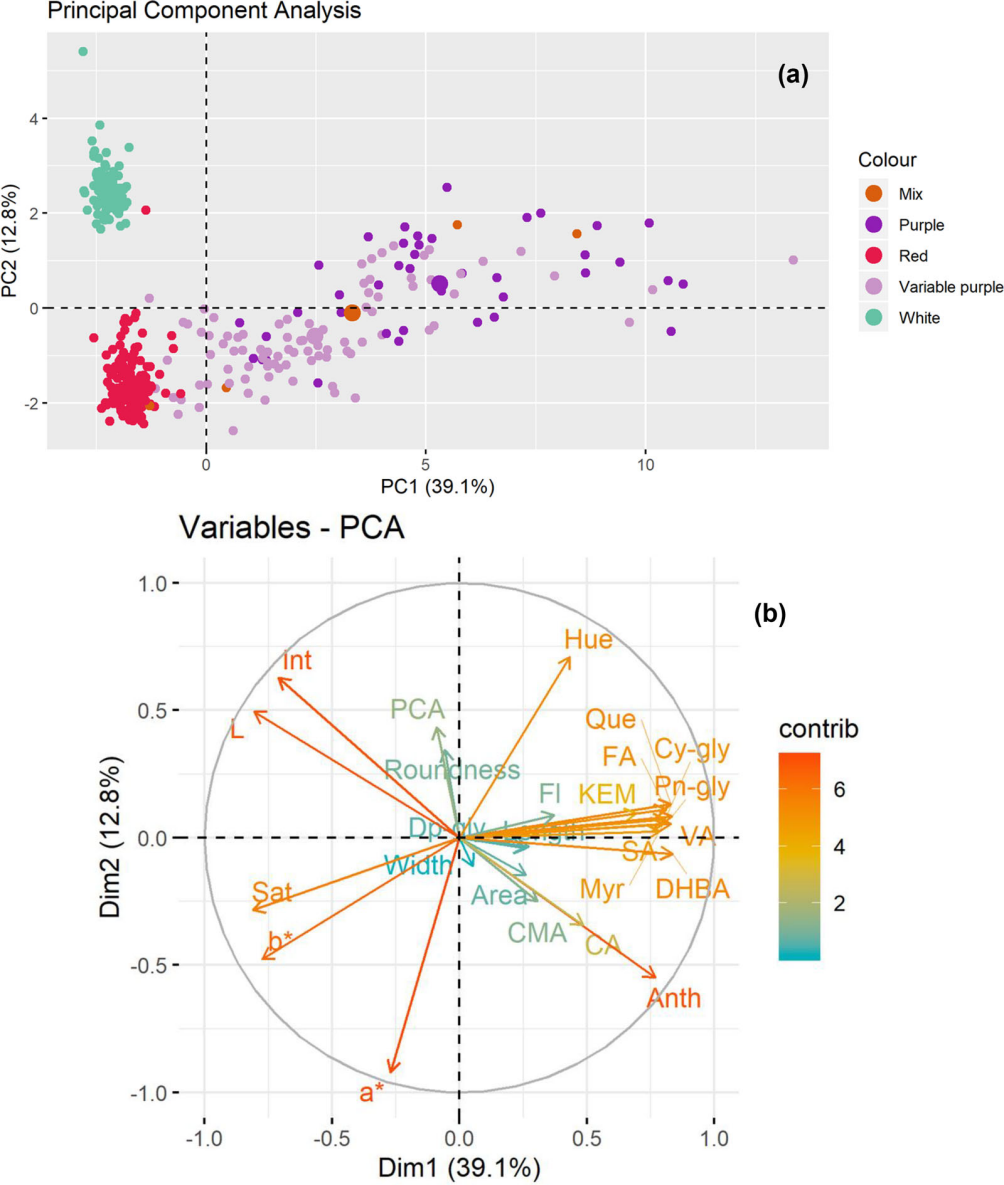
Principal component analysis (PCA) of the 364 retained rice accessions using geometric, color‐related and nutritional traits. (A) PCA showing the distribution of the 364 rice accessions relative to visual color assessment. (B) PCA trait distribution and contribution to the total variance for the Videometer parameters saturation (Sat), Cielab_L (*L*), Cielab_A (a*), Cielab_B (b*), hue, intensity (Int), and anthocyanin (Anth), and the nutritional traits protocatechuic acid (DBHA), peonidin glycoside (Pn‐gly), quercetin (Que), kaempferol (KEM), cinnamic acid (CA), syringic acid (SA), vanillic acid (VA) ferulic acid (FA), and cyanidin glycoside (Cy‐gly).

PCA of the geometric, color‐related and nutritional traits separated the accessions into three main groups (Figure 3A). The first group contained white accessions and the second group contained red accession, while the third group contained mix, purple, and variable purple accessions. Considerable phenotypic variation was observed among variable and purple rice accessions. The first principal component (PC) explained 39.1% of the phenotypic variation, while PC2 explained 12.8%. The variation in PC1 was mainly associated with nutritional traits, while variation in the PC2 was driven by color‐related traits (Figure 3B).

### 3.2 SNPs discovery and quality control

The unfiltered hapmap file obtained from Elshire Group Ltd. contained 558,526 SNP markers. After removing SNP with CR <0.9 and MAF <0.05, a total of 81,580 SNP markers were retained. The distribution of the ratio Ho/He showed a binomial distribution (Figure S6). Using a cut‐off value of Ho/He < 0.5, heterozygous SNPs markers were filtered out, leaving 64,139 SNPs markers for subsequent analyses (Figure S7). The number of markers on each chromosome varied from 3,478 on chromosome 12 to 8,756 markers on chromosome 1, with an overall marker density of 1 marker every 5.93 kb (Table S5, Figure S8). Filtering resulted in 64,139 high‐quality markers that were used for the GWAS. Twelve rice accessions with a heterozygosity rate of above 10% were removed, leaving 364 rice accessions for subsequent analyses (Table S1). A selection of 5,181 SNP markers, which reduced the influence of strong LD, were retained for population structure and genetic diversity assessment.

When considering all 364 rice accessions, the LD decay distance was 350.58 kb at *r* ^2^ = 0.2. For *indica* accessions, the value of *r* ^2^ decreased to 0.2 at about 223.29 kb, while in *japonica* the value *r* ^2^ decreased to 0.2 at about 189.64 kb (Figure S9). The LD decay varied across chromosomes, with the shortest LD decay distance of 105.34 kb being seen with chromosome 5, and the longest distance of 498.35 kb with chromosome 1 (Table S5).

### 3.3 Genetic diversity within the rice accessions

High genetic diversity was present within the 364 accessions (He = 0.24 ± 0.12; Wilcoxon signed rank test, *p* < 0.05), with higher levels of diversity being observed within the *indica* (He = 0.24 ± 0.18) compared to *japonica* (He = 0.18 ± 0.18) rice accessions (Table 1). Within grain color groups, the highest diversity was observed in red rice accessions (He = 0.25 ± 0.13), followed by purple (He = 0.24 ± 0.14) and then non‐pigmented (He = 0.22 ± 0.14). The level of inbreeding was high across all groups (He = 0.96 ≤ FIS ≥ 0.97) (Table 1).

**TABLE 1:** Summary statistics of genetic diversity indicators across rice subspecies and seed color groups.

		A ± SD	Percentage	Ar ± SD	Ho ± SD	He ± SD	Fis	Fis_Low	Fis_High
Subspecies groupings	*Indica*	9444 ± 0.38	91.14	1.79 ± 0.38	0.01 ± 0.03	0.24 ± 0.18	0.96	0.95	0.97
	*Japonica*	9139 ± 0.42	88.2	1.7 ± 0.42	0.01 ± 0.02	0.18 ± 0.18	0.96	0.95	0.97
	*temperate japonica*	7840 ± 0.50	75.68	1.47 ± 0.47	0.01 ± 0.03	0.13 ± 0.18	0.95	0.92	0.96
	*Tropical japonica*	8849 ± 0.45	85.4	1.59 ± 0.45	0.01 ± 0.02	0.16 ± 0.18	0.96	0.95	0.97
Color	Purple	10328 ± 0.08	99.67	1.98 ± 0.10	0.01 ± 0.02	0.24 ± 0.14	0.97	0.96	0.98
	Red	10356 ± 0.03	99.94	1.99 ± 0.04	0.01 ± 0.02	0.25 ± 0.13	0.97	0.97	0.98
	White	10311 ± 0.1	99.51	1.97 ± 0.12	0.01 ± 0.02	0.22 ± 0.14	0.96	0.93	0.97

*Note*: Total number of alleles observed across SNP marker loci (A). Total observed alleles per locus as a percentage of population samples (%). Mean allele richness (Ar). Observed heterozygosity across loci (Ho). Expected heterozygosity across loci (He). Inbreeding coefficient (FIS) showing the 95% confidence intervals (FIS_Low and FIS_High).

An *F*_ST_ test was used to assess genetic differentiation between all groups based on allele discrepancies. Genetic differentiation was high between *indica* and *japonica* (*F*_ST_ = 0.31), between *indica* and *temperate japonica* (*F*_ST_  =  0.36), *tropical japonica* and *indica* (*F*_ST_  =  0.33), and between *tropical* and *temperate japonica* (*F*_ST_  =  0.29) groups (Table 2). When accessions were assessed based on pericarp coloration, little differentiation was observed between the different groups (*F*_ST_ < 0.05) (Table 2).

**TABLE 2:** Pairwise *F*_ST_ comparison between rice subspecies and seed color groups.

	Populations	*F*_ST_	Lower	Upper
Subspecies groupings	*Indica* vs. *japonica*	0.31	0.30	0.31
	*Indica* vs. temperate *japonica*	0.36	0.34	0.38
	*Indica* vs. tropical *japonica*	0.33	0.32	0.33
	Temperate *japonica* vs. tropical *japonica*	0.29	0.26	0.31
Color groupings	Purple vs. red	0.02	0.01	0.04
	Purple vs. white	0.03	0.02	0.05
	Red vs. white	0.02	0.01	0.05

AMOVA indicated that 30.30% of the total genetic variation occurred between the *indica* and *japonica* groups, while within group differences accounted for 67.53% of the variation. Residual, within rice accession heterozygosity, accounted for 2.18% of the total genetic variation. Comparing groups within sub‐species, the molecular variance increased to 32.49%, while between the accessions within those groups, the variation was 65.26% (Table 3). When rice color groups were compared, most of the genetic variation was within the groups (95.02%), with very little between group variation (2.45%), indicating that diversity for pericarp color was equally as high in *indica* and *japonica* subspecies groups. The remaining variation was within rice accessions (2.53%) (Table 3).

**TABLE 3:** Analysis of molecular variance (AMOVA) between and within rice subspecies and seed color groups.

	Source of variation	Degree of freedom	Sum of squares	Mean sum of square	Estimate variance	Percentage of variation
Sub‐species	Between groups	1	99,218.84	99,218.84	299.86	30.30
	Between accessions within group	349	474,011.20	1358.20	668.33	67.53
	Within accessions	351	7557.52	21.53	21.53	2.18
	Total	701	580,787.57	828.51	989.72	100
Color	Between groups	2	12,438.21	6219.10	19.69	2.45
	Between accessions within group	356	550,759.52	1547.08	763.38	95.02
	Within accessions	359	7290.82	20.31	20.31	2.53
	Total	717	570,488.55	795.66	803.38	100
Subspecies groups	Between groups	2	132,656.7	66,328.33	309.75	32.49
	Between accessions within groups	348	440,573.4	1266.02	622.242	65.26
	Within accessions	351	7557.52	21.53	21.53	2.26
	Total	701	580,787.6	828.51	953.53	100

### 3.4 Population structure within the 364 retained rice accessions

**FIGURE 4:**
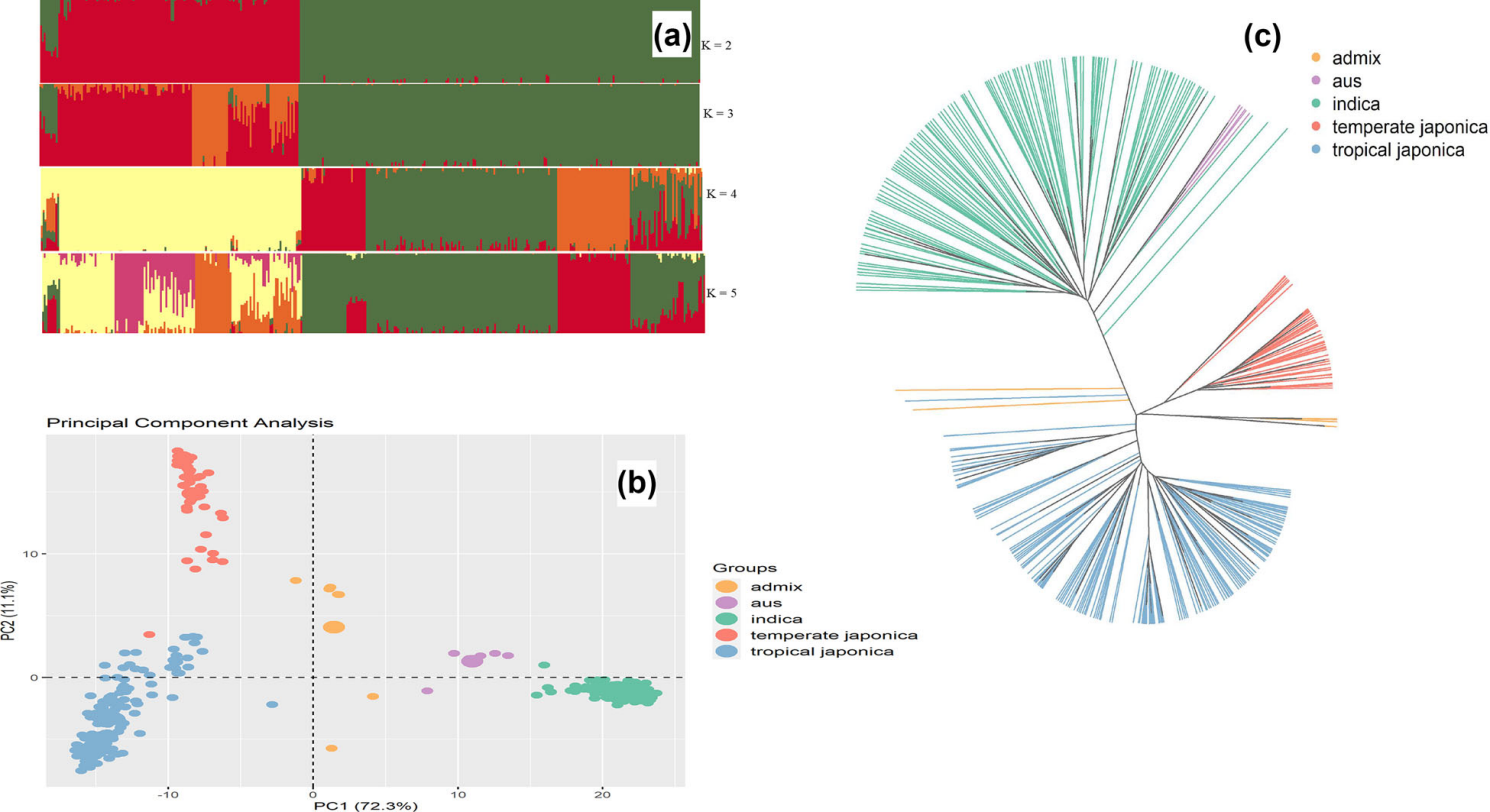
Analysis of the genetic relationship between the 364 retained rice accessions, applying 5,181 independent single nucleotide polymorphism (SNP) markers. (A) Structure analysis. Each individual accession is represented by a vertical line. The proportion of the color of each vertical line represents the proportion contributed by the ancestral population. The best supported clustering (*K* = 2) divided the 364 rice accessions into two main groups, corresponding to *indica* and *japonica* rice types. With increased *K* values (*K* = 3, 4, and 5), additional substructure within each cluster was observed. (B) Principal component analysis (PCA). The additive relationship matrix was calculated in rrBLUP using the R package rrBLUP (Endelman, 2011) and used as input to the PCA. (C) Neighbor‐joining tree, constructed based on bitwise genetic distance.

The population structure of the 364 rice accessions was evaluated using 5,181 independent SNP markers. Structure analysis indicated the presence of the two main rice subspecies, *indica* and *japonica*, as well as subdivisions within each (Figure 4A). The majority of the rice accessions belonged to the *japonica* sub‐species (60.71%), while 36.54% were classified as *indica*, leaving 2.75% of accessions as admixed (Table S1). PCA supported the ADMIXTURE results, with a clear separation between *japonica* and *indica* types (Figure 4B). Subdivisions were identified within each subspecies. *Japonica* accessions were separated into *tropical japonica* and *temperate japonica*, and a subset of *indica* accessions was classified as *aus* (Figure 4B). While most of the accessions from the Philippines and Indonesia were *tropical japonica*, and most of the accessions in the *temperate japonica* sub‐group originated from Thailand, Myanmar, Cambodia, Thailand, and Lao Pdr, PCA did not suggest a relationship between geographical origin and genetic structure (Figure S10). The neighbor‐joining tree analysis (Figure 4C) produced similar results to those obtained with PCA and ADMIXTURE.

### 3.5 Genome‐wide marker trait associations

A total of 64,139 polymorphic markers with a call rate ≥0.9 and MAF ≥0.5 were used to identify genomic regions associated with the geometric, color‐related and nutritional parameters measured in the retained 364 rice accessions. Two MLM approaches were utilized: the single‐locus MLM method developed by EMMAX (Kang et al., 2010) and the MLMM (Segura et al., 2012), which assumes that multiple loci are associated with the phenotype. Manhattan plots from the EMMAX and MLMM analyses are shown in Figure S11.

Using EMMAX and the nominal threshold (*p* ≤ 1 × 10^−5^) as the cut‐off for significance, a total of 743 SNPs, corresponding to 423 unique loci, were identified for 20 of the 25 traits (Table 4, Table S6). The number of MTAs varied from 2 for the color‐related traits *L* and anthocyanin to 204 for ferulic acid. Using MLMM, 93 SNPs, corresponding to 70 unique loci, were detected. The number of MTAs varied from 1 for width and roundness to 9 for ferulic acid (Table 4, Table S7). After applying the Bonferroni correction‐value of −log10(*P*) = 6.1, EMMAX identified 401 significant SNPs located within 228 putative loci (Table 4, Table S8). Using the MLMM, 53 significant SNPs, corresponding to 40 loci, were found associated with 16 traits (Table 4, Table S9). No MTAs were reported for the multi‐spectral imaging trait intensity, or the phenolic compounds flavone, p‐coumaric acid, cyanidin glycoside, and delphinidin glycoside using any of the above approaches. Therefore, these nutritional phenotypes were scored as a binary phenotype, that is, the phenolic compound either being detected (present) or not detected (absent). Applying the Bonferroni correction, this approach detected 12 MTAs using EMMAX and MLMM (Table S10).

**TABLE 4:** Summary of the marker trait associations (MTAs) detected by applying a single locus mixed‐linear model implemented in Efficient Mixed‐Model Association eXpedited Model (EMMAX) and the multi‐locus linear mixed model (MLMM).

Trait	EMMAX nominal threshold (Table S6)	Chrom.	MLMM nominal threshold (Table S7)	Chrom.	EMMAX Bonferroni correction (Table S8)	Chrom.	MLMM Bonferroni correction (Table S9)	Chrom.
Area	3	2 and 12	3	2 and 12	0	n/a	0	n/a
Length	26	3,5, and 8	5	3,5,7, and 8	5	3	1	3
Width	6	5	1	5	5	5	1	5
Roundness	17	3	1	3	4	3	1	3
Saturation	43	2,3,4,7 and 10	4	2,3, and 7	25	2,3,4, and 7	4	2,3, and 7
Cielab_A	64	1 and 7	6	2,7 and 10	47	7	1	7
Cielab_B	75	1,2,3,4, and 7	6	1,2,3,7, and 9	42	1,2,3,4, and 7	5	1,2,3, and 7
Cielab_L	2	3	2	3	0	n/a	0	n/a
Hue	50	7	5	2,6, and 7	39	7	1	7
Intensity	0	n/a	0	n/a	0	n/a	0	n/a
Anthocyanin	2	3	2	1 and 3	0	n/a	2	1 and 3
Ferulic acid	204	1,2,3,4,5,6,8,9,10, and 12	9	1,2,3,4,5, and12	112	1,2,3,4,5,8,10, and 12	8	1,2,3,4,5, and 12
Vanillic acid	48	1,4, and 7	8	1,3,4, and 8	17	4	1	4
Kaempferol	25	1,2,3,4,5, and 7	8	1,2,3,7,9, and11	11	1,2,3, and 4	7	1,2,3,7,9, and 11
Quercetin	80	1,2,3,4,8,10,11, and 12	7	2,3,4,5,10, and 12	31	1,2,3,4,8,10,11, and 12	6	2,3,4,5,10, and 12
Syringic acid	12	4,7, and 9	5	5,6,7, and 9	6	7	4	5,6,7, and 9
Peonidin glycoside	13	1,3,4,6, and 10	8	1,2,3,4,6, and 9	7	3,4, and 6	8	1,2,3,4,6 and,9
Protocatechuic acid	14	2,3, and 4	3	2 and 3	4	2 and 3	1	2
Catechin	51	7	2	7	46	7	2	7
Cinnamic acid	5	3 and 7	5	3 and 7	0	n/a	0	n/a
Myricetin	3	3,4, and 10	3	3,4, and 10	0	n/a	0	n/a
p‐Coumaric acid^a^	0	n/a	0	n/a	2	1	2	1
Flavone^a^	0	n/a	0	n/a	0	n/a	1	3
Cyanidin glycoside^a^	0	n/a	0	n/a	7	3 and 4	2	3 and 4
Delphinidin glycoside^a^	0	n/a	0	n/a	0	n/a	2	1 and 3
Total	743		93		410		60	

^a^ MTA for these nutritional traits were only identified when assessing the data set as phenolic compound present or absent (Table S10). Abbreviations: Chrom., chromosome number; n/a, not applicable.

Significant SNPs associated with grain length on chromosome 3 explained 6.60%–7.92% of the phenotypic variance (Tables S6–S8). The most significant SNP (S03_16840706) was located at the 5′ UTR of LOC_Os03g29540, which encodes for an ATP‐dependent protease. SNPs significantly associated with roundness were also found on chromosome 3, accounting for 7.55% of the phenotypic variance (Tables S6–S8). The most significant SNP (S03_16725803) was within an intergenic region between loci LOC_Os03g29370 and LOC_Os03g29389, both of which are annotated as hypothetical proteins without a known functional. Five SNPs were associated with width on chromosome 5, accounting for 7.01%–10.78% of the phenotypic variance (Table S8). We identified 25 SNPs significantly associated with saturation on chromosomes 2, 3, 4, and 7 that explained 6.78%–11.51% of the phenotypic variance (Table S8). For a*, 47 significant SNPs were detected on chromosome 7, while 42 SNPs associated with b* were located on chromosomes 1, 2, 3, 4, and 7 (Table S8). The phenotypic variance explained by the individual SNPs ranged from 6.53% to 16.16%. Thirty‐nine SNPs were identified on chromosome 7 associated with hue, explaining 10.51%–19.89% of the phenotypic variation (Table S8).

Six significant SNPs associated with syringic acid were found on chromosome 7 (Table S8). The most significant SNP (S07_6090597) was a synonymous SNP within the rice gene *OsIPT5*, which encodes for the isopentenyl transferase 5 protein (Nguyen et al., 2021). SNP S07_6090597 was also significantly associated with the color‐related traits a*, b*, saturation and hue, and catechin levels (Table S8). Forty‐six SNPs were associated with catechin on chromosome 7 (Table S8) with the most significant SNP S07_6069227, which explained 26.06% of the phenotype variance, being located at the 3′ UTR of the *Rc*/*OsbHLH17* gene. With ferulic acid, 112 significant SNPs were identified across chromosomes 1, 2, 3, 4, 5, 8, 10 and 12, with phenotypic variance values ranging from 6.68% to 18.11% (Table S8). The most significant SNPs laid within predicted genes peroxidase 2, stress repressive zinc finger protein 3, grain weight 2, DnaJ domain protein C30, and spotted leaf 11. A total of 17 MTAs were found for vanillic acid on chromosome 4 (Table S8). The most significant SNP, explaining 11.37% of the phenotypic variance, was located upstream of the *Subtilisin 44* gene involved in protein degradation. The 31 SNPs associated with quercetin were identified on eight chromosomes and explained 7.93% to 15.69% of the phenotypic variance (Table S8). Two of the most significant SNPs (S02_8132119 and S01_8132119), explaining 15.69% and 14.61% of the phenotypic variance of quercetin, were located in the intergenic position between the gene *grain weight 2* and LOC_Os02g14730, and within an intronic regions of *OsGSTF7*, respectively. Eleven SNPs were found to be associated with kaempferol (Table S8). These SNPs were located on chromosomes 1, 2, 3, and 4 and explained between 6.70% to 8.92% of the phenotypic variance. MTAs associated with protocatechuic acid were located on chromosomes 2 and 3, and explained 7.93% to 9.70% of the phenotypic variance (Table S8). The most significant SNP on chromosome 2 (S02_8132119) was located within an intergenic region between a *Grain Weight 2* gene and a gene encoding for a ubiquitin carboxyl‐terminal hydrolase family protein. On chromosome 3, SNP S02_10771077 was located downstream of LOC_Os03g19200, within an exon of *OsALS*, which encodes for acetolactate synthase. With peonidin glycoside, seven significant SNPs were detected on chromosomes 3, 4, and 6, with phenotypic variance ranging from 8.79% to 13.47% (Table S8). The most significant SNP (S03_16672065) lies within an exon of LOC_Os03g29280.

Significant MTAs were not found for myricetin and cinnamic acid when applying the Bonferroni correction, but only when using the nominal threshold (Table S7). Assessing the data as a binary phenotype, seven statistically significant associations were detected between SNP markers and cyanidin glycoside located on chromosomes 3 and 4, explaining 7.0% to 9.58% of the phenotypic variance (Table S10). SNP associations with p‐coumaric acid were found on chromosome 1 and explained 7.09% to 9.92% of the phenotypic variance (Table S10). A single MTA was found for flavone on chromosome 1, while two MTAs were found for delphinidin glycoside on chromosomes 1 and 3 (Table S10).

The MLMM approach resulted in fewer significant hits between SNPs and phenotypic traits, using both the nominal and Bonferroni thresholds (Tables S7 and S9), compared to EMMAX (Tables S6 and S8). However, MLMM did identify MTA that were not detected by EMMAX. A significant SNP on chromosome 7 (S07_2177220) was found to be associated with the color‐related traits saturation and b*, while an MTA was found for b* on chromosome 1 (S01_15126251) (Table S9). An MTA was found for anthocyanin on chromosome 1 (S01_28051821) (Table S9). This SNP was located in the 5′ UTR of a wax synthase gene, LOC_Os01g48874. Numerous MTAs were found for ferulic acid when applying EMMAX with the Bonferroni correction (Table S8); however, only eight significant SNPs were identified to be associated with ferulic acid levels when applying MLMM and the Bonferroni correction (Table S9), including four SNPs that were not detected by EMMAX. Four MTAs were found for kaempferol on chromosomes 3, 7, 9, and 11, and three MTAs for quercetin on chromosomes 4 (S04_1780371), chromosome 10 (S10_13843153), and chromosome 12 (S12_9926677) (Table S9). The SNP on chromosome 12 was located within LOC_Os12g17340, which encodes for a CC‐NBS‐LRR resistance protein MLA13. The SNP on chromosome 4 was also associated with ferulic acid (Table S8). Two MTAs were found for syringic acid on chromosomes 5 and 6, located in LOC_Os05g31525 and LOC_Os06g03676 (Table S9). An additional MTA was found for catechin on chromosome 7 (S07_6259359) that was also associated with the color‐related traits hue and a* (Table S7). Four MTAs were found for peonidin glycoside, two on chromosome 9 (S09_21842414 and S09_5624836), one on chromosome 3 (S03_2052290), and a fourth on chromosome 1 (S01_28597777), which was also associated with kaempferol (Table S6).

### 3.6 Co‐localization of marker trait associations between traits

Many of the significant SNPs were associated with more than one trait (Tables S11–S14). Most colocalization occurred between nutritional traits, and nutritional and color‐related traits. Many of the multi‐trait loci involved MTA for ferulic acid, quercetin, and kaempferol. In addition, some of these loci were also associated with b*. A large number of multi‐trait loci were found that linked catechin levels with the color‐related traits hue, a* and b*, as well as saturation and syringic acid. Links were also found between vanillic acid and saturation and b*, and between peonidin glycoside and saturation and b*. The SNP S07_6069227, located in the 3′ UTR of the *RC/OsbHLH17* gene, was associated with catechin and syringic acid, and the color‐related traits hue, saturation, a*, and b*. The SNP S03_16672065, located within LOC_Os03g29280 (hypothetical protein), was linked with saturation, b*, and peonidin glycoside. The SNP S02_8132119 was associated with quercetin, ferulic acid and protocatechuic acid, and the color‐related traits saturation and b*. S02_8132119 is located between the rice gene *OsGW2*, which encodes for Grain Width and Weight2, and LOC_Os02g14730. *OsGW2* represses cell expansion in rice endosperm, affecting grain filling, weight, width, and yield (Song et al., 2007).

### 3.7 Association network of targeted multi‐spectral imaging and nutritional data

**FIGURE 5:**
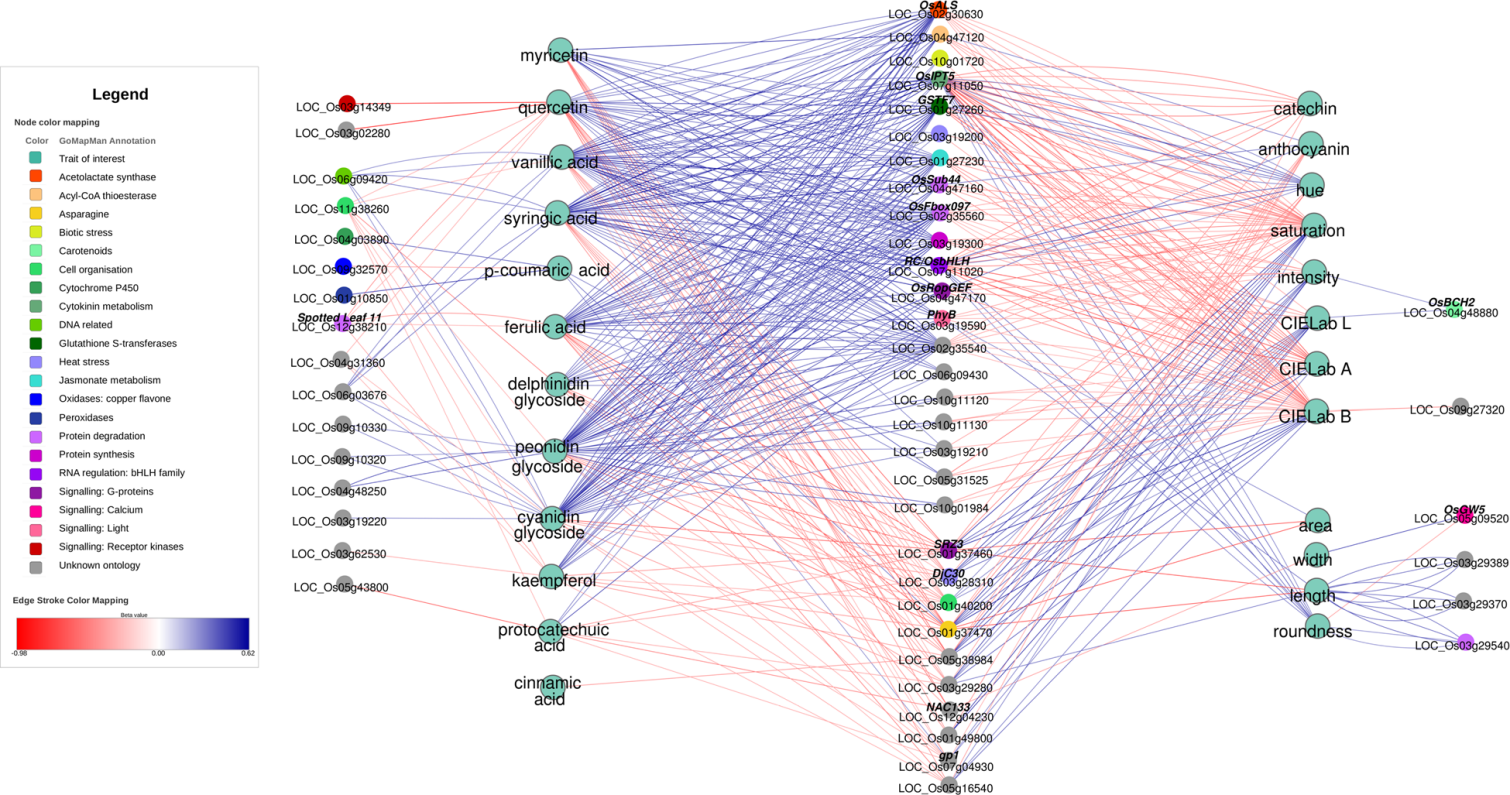
Association network summary of candidate genes linked with rice grain geometric, color‐related and nutritional traits. Targeted association analysis identified 520 significant single nucleotide polymorphism (SNP)–trait interactions with high beta effects (−0.3 > *β* or *β* > 0.3), which involved 67 unique SNPs, within 52 candidate genes, associated with 24 traits of interest.

Targeted association analysis identified 520 significant SNP–trait interactions with high beta effects (−0.3 > *β* or *β* > 0.3), which involved 67 unique SNPs, within 52 candidate genes, that were associated with 24 traits of interest (Figure 5, Figure S12, Table S15). The reference alleles of 14 candidate genes with positive beta effects associated with nutritional traits displayed negative beta effects with color‐related traits. Four SNPs within an acetolactate synthase gene (*OsALS*) showed positive beta effects for cyanide glycoside, ferulic acid, peonidin glycoside, quercetin, syringic acid, and vanillic acid but negative beta effects with b* and saturation. Similarly, the candidate gene *OsIPT5* showed positive effects with cyanidin glycoside, peonidin glycoside, syringic acid, and vanillic acid but negative effects with saturation, a*, and b*. The reference alleles of three significant SNPs within the gene *Rc/bHLH17* showed positive effects for syringic acid and vanillic acid but negative effects for saturation, a*, and b*. Both candidate genes *OsIPT5* and *Rc/bHLH17* exhibited strong associations with catechin content. The candidate gene *OsGSTF7*, which encodes a glutathione S‐transferase, contained two intronic SNPs positively associated with cyanidin glycoside, ferulic acid, kaempferol, myricetin, peonidin glycoside, quercetin, syringic acid, and vanillic acid but showed negative effects with a*, b*, and saturation. Similarly, two candidate genes involved in protein degradation, *OsSub44* (Subtilisin 44, with potential serine‐type endopeptidase activity; Zheng et al., 2022) and *OsFbox97* (a cyclin‐like F‐box domain containing protein 97; Hua et al., 2011) exhibited positive effects with nutritional traits but negative effects with color‐related traits.

Nine candidate genes, having SNPs positively affecting color‐related traits but negatively affecting nutritional traits, included *OsSRZ3* (C. Zhang et al., 2020), which positively affected saturation, a*, b*, and *L*, but negatively affected cyanidin glycoside, ferulic acid, kaempferol, peonidin glycoside, quercetin, and vanillic acid levels. Similarly, *OsDiC30* (a DnaJ domain protein C30) was negatively associated with anthocyanin, cyanidin glycoside, protocatechuic acid, ferulic acid, myricetin, peonidin glycoside, quercetin, syringic acid, and vanillic acid, while showing positive effects with b*, *L*, intensity, and saturation.

The association network analysis also helped to identify candidate genes unique to specific phenolic compounds. LOC_Os03g14349, a Ty1‐copia subclass retrotransposon and LOC_Os03g02280, a DUF584 domain containing protein, were associated with quercetin. An upstream SNP of LOC_Os03g62530, a conserved gene with unknown function, strongly associated with kaempferol, while protocatechuic acid was associated with the predicted gene LOC_Os05g43800 and cinnamic acid with an exonic SNP of LOC_Os05g38984. With grain geometric traits, the known gene *OsGW5* was positively associated with width but negatively associated with roundness. Grain length was also associated with SNPs from three loci on chromosome 3: LOC_Os03g29540, LOC_Os03g29389, and LOC_Os03g29370.

### 3.8 Traits associated with *Rc/bHLH17* and *IPT5* gene haplotypes

Significant SNPs found within the 3′UTR and exonic regions of the *Rc/bHLH17* gene identified four haplotypes, each contributing to distinct phenotypes (Figure S13). Rice accessions with the haplotype AGC were mostly red, while the rice accessions with the TAT haplotype varied in grain color. Accessions carrying the TAT haplotype in general had higher levels of cinnamic acid, cyanidin glycoside, kaempferol, myricetin, p‐coumaric acid, peonidin glycoside, quercetin, and vanillic acid, while AGC haplotype accessions showed higher a*, b*, and saturation values. However, AGC rice accessions had higher levels of catechin compared to TAT accessions. The other two haplotypes, AAT and TGC, were rare haplotypes represented by only nine and three rice accessions, respectively.

The four haplotypes found by five significant SNPs located within exonic regions of the *IPT5* gene also showed distinct phenotypes (Figure S14). The haplotype CGCCT showed lower a*, b*, saturation and catechin levels, but higher levels of cyanidin glycoside, ferulic acid, flavone, quercetin, vanillic acid, myricetin, p‐coumaric acid, peonidin glycoside, and syringic acid, as well as less roundness compared to other haplotype groups.

**FIGURE 6:**
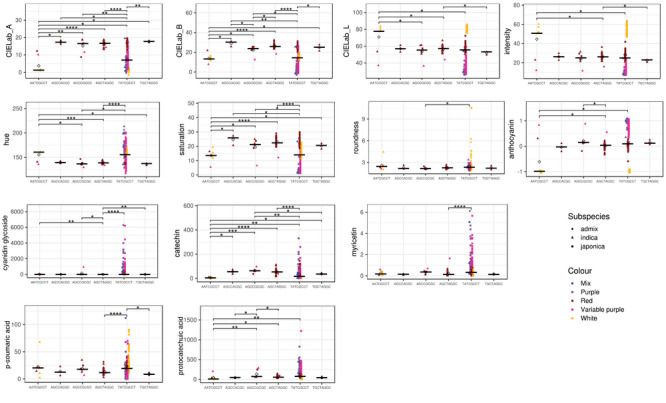
Haplotype analysis of significant single nucleotide polymorphisms (SNPs) identified in *Rc/bHLH* and *IPT5* genes. Significant SNPs within the genes *Rc/bHLH17* and *OsIPT5* showed functional haplotypes explaining variation within geometric, color‐related, and nutritional‐related traits of the 364 rice accessions.

Combining SNPs from *Rc/bHLH17* and *IPT5* generated six haplotypes with distinct phenotypes (Figure 6). Most of the rice accessions had haplotype TATCGCCT and included rice lines of all grain color groups. Haplotype TATCGCCT had significantly higher levels of cyanidin glycoside, myricetin, and p‐coumaric acid but lower levels of catechin than haplotype AGCTAGGC, as well as low saturation, a* and b* levels, and high hue compared to the red accessions within haplotype AGCTAGGC. Haplotype AATCGCCT also differed from the other haplotypes, having lower saturation, a*, b*, anthocyanin, and catechin values and high values of *L* and intensity.

## 4 DISCUSSION

The international rice genebank maintained by IRRI hosts an untapped reservoir of rice accessions with varying pericarp coloration, providing a wealth of biodiversity for rice nutritional breeding. By targeting specific phenolic compounds, with known nutritional potential, this study was able to identify rice accessions, candidate genes and specific haplotypes of value for the enhancement of the nutritional potential of new rice varieties. The identification of multi‐trait loci, that contribute to both pericarp color‐related parameters and the level of valuable phenolic compounds, provides the opportunity to select for nutritionally valuable rice accessions based on these color‐related parameters.

GWAS is a powerful tool to identify genetic variation associated with traits. Using a panel of 364 accessions and 64,139 SNPs markers, significant genomic regions associated with geometric, color‐related, and nutritional‐related traits were identified. Many of the significant SNPs were associated with more than one trait, with most colocalization occurring between nutritional traits, and between nutritional and color‐related traits. The colocalization of trait loci aligned well with the correlations seen between traits in the Pearson correlation and network visualization analyses. The association networks, based on the identified candidate genes, further emphasized the interconnectivity between traits, highlighting the genetic linkage between pericarp pigmentation and secondary metabolites.

One of the most significant SNPs, S07_6069227, was located in the 3′ UTR of the gene *Rc/bHLH17* and associated with catechin and syringic acid, and the color‐related traits hue, saturation, a*, and b*. Being a positive regulator of proanthocyanidin, *Rc/bHLH17* is known to play a key role in regulating pericarp color in rice (Sweeney et al., 2006). The targeted association analysis further identified positive beta effects for the gene *OsIPT5*, which encodes for an isopentenyl transferase and has previously been suggested to play a role in pericarp color and metabolite accumulation (Brotman et al., 2021). Positive beta effects were found between *OsIPT5* and cyanidin glycoside, peonidin glycoside, syringic acid, and vanillic acid, as well as with roundness and hue, while negative beta effects were found for *OsIPT5* with a*, b*, and saturation.

The six haplotypes identified from significant SNPs within the genes *Rc/bHLH17* and *OsIPT5* identified a range of phenotypes, particularly for color‐related traits (Figure 6). The genes *Rc/bHLH17* and *OsIPT5* have previously been suggested to play a role in catechin accumulation, pericarp color determination, starch structural composition, glycemic index values, and antioxidant metabolites (Brotman et al., 2021). The haplotypes identified in this study were similar to the haplotypes identified by Brotman et al. (2021), of which haplotype 4 (GATGCGACCAGAGTTAGAGGTGT) exhibited antioxidant activity, contributing to increased anti‐cancer properties, as demonstrated using cancer cell lines. However, in this study, phenotypic variation between the six haplotypes was observed not only for catechin but also for protocatechuic acid, anthocyanin, cyanidin glycoside, myricetin, and p‐coumaric acid, suggesting that *Rc/bHLH17* and *OsIPT5* may have an important role in the regulation of a wider range of phenolic compounds, and not only those directly conferring pericarp color.

Aside from *Rc/bHLH17* and *OsIPT5*, multiple candidate genes, not previously reported, were found to be linked to the color‐ and nutritional‐related traits analyzed. These genes fell into two groups, those that conferred positive effects toward nutritional traits, but negative effects towards color‐related traits, and those that conferred positive effects toward color‐related traits, but negative effects toward nutritional traits. It is evident that both groups of candidate genes could be key regulators of pigmentation, as well as metabolite profiles in rice. Moreover, color‐parameters such as a*, b*, L*, saturation, and intensity could be used as indicators of the presence or abundance of the associated phenolic compounds in rice.

Some of the candidate genes included Os*PhyB*, *OsGSTF7*, *OsALS*, *OsSub44*, *OsRopGEF*, *OsSRZ3*, and *OsBCH2*. The candidate gene *OsPhyB* encodes for a phytochrome B protein, which is the main photoreceptor for red light (Nagy & Schafer, 2002) and interacts with *bHLH* transcription factors to regulate red/far‐red light phototransduction (Rausenberger et al., 2010). Previously, it has been noted that the biosynthesis of anthocyanin and proanthocyanidin is influenced by high light intensity (Ma et al., 2018; Y. Zhang et al., 2018), supporting the observations of rice grain having deeper pigmentation when grown at higher elevations. The gene *OsGFT7* encodes for a glutathione S‐transferase (GST). The family of GST genes is known to be involved in detoxification of xenobiotics (Armstrong, 1997), regulation of redox homeostasis for cell protection against UV radiation and oxidative stress (Jiang et al., 2010), herbicide response (Cummins et al., 2013), as well as biosynthesis and transport of secondary metabolites (Dixon et al., 2010). *OsALS* encodes for an acetolactate synthase, a thiamine pyrophosphate enzyme involved in the first biosynthetic step in branched‐chain amino acid synthesis, catalyzing the reaction of two pyruvate molecules to produce acetolactate, which is known as an intermediate product of valine, leucine, and isoleucine (Yean et al., 2021). *OsSub44* encodes for a putative subtilisin homologue having orthologues functions with cucumisin and/or xylem serine proteinase 1. These proteinases are known to be involved in pathogen response and programmed cell death in plants (Vartapetian et al., 2011). *OsRopGEF* encodes for a rho guanine nucleotide exchange factor whose orthologue in *Arabidopsi*s, ATROPGEF7, is involved in pattern formation during the formation of secondary cell wall pits (Nagashima et al., 2018). The candidate gene *OsSRZ3*, which was negatively associated with the phenolic compounds kaempferol, quercetin, ferulic and vanillic acids, and cyanidin and peonidin glycosides, but positively associated with the color‐related traits a*, b*, *L*, and saturation, encodes for a stress repressive zinc finger protein. It belongs to a gene family where the genes contain at least one highly conserved SRZ domain and are known to be involved in abiotic stress responses (C. Zhang et al., 2020). The color‐related traits intensity and L* were positively associated with the candidate gene *OsBCH2*, which encodes a beta‐carotene hydroxylase 2 involved in the hydroxylation of beta‐carotene, producing zeaxanthin, known to be important in the regulation of the xanthophyll cycle in plants and adaptation to high light stress (Johnson et al., 2008). Zeaxanthin, together with lutein, comprises more than 90% of the total carotenoids found in rice (Ashraf et al., 2017; Melini & Acquistucci, 2017).

The development of markers that identify the *Rc/bHLH17* and *IPT5* haplotypes associated with enhanced nutritional value would provide robust tools for selecting these positive alleles at early stages within a rice breeding program, eliminating the need for extensive phenotyping. Bi‐parental mapping of informative crosses would further validate many of the additional MTAs and candidate genes identified in this study. These candidate genes, interlinking color‐related traits with phenolic content, provide useful targets for future experimental studies, including haplotype analysis. The relevant haplotypes could then be used to screen further rice accessions to select appropriate rice donor lines to breed for new rice varieties with enhanced nutritional value. The associations between phenolic compound content and color‐related traits could also enable nutritionally valuable rice genotypes to be identified quickly through videometer analysis.

## AUTHOR CONTRIBUTIONS

**Edwige Gaby Nkouaya Mbanjo**: Data curation; formal analysis; investigation; methodology; resources; validation; writing—original draft; writing—review & editing. **Erstelle A. Pasion**: Formal analysis; writing—original draft; writing—review & editing. **Huw Jones**: Formal analysis; investigation; methodology; writing—original draft; writing—review & editing. **Socorro Carandang**: Methodology; writing—review & editing. **Gopal Misra**: Formal analysis; writing—review & editing. **John Carlos Ignacio**: Data curation; formal analysis; writing—review & editing. **Tobias Kretzschmar**: Conceptualization; data curation; investigation; project administration; supervision; writing—review & editing. **Nese Sreenivasulu**: Conceptualization; data curation; project administration; resources; supervision; writing—review & editing. **Lesley Ann Boyd**: Conceptualization; funding acquisition; investigation; project administration; supervision; writing—original draft; writing—review & editing.

## CONFLICT OF INTEREST STATEMENT

The authors declare no conflict of interest.

## Supporting information

Figure S1 Classification of the 364 retained rice accessions based on country‐of‐origin. X‐axis = the country‐of‐origin. Y‐axis = the number of accessions from each country.

Figure S2 Classification of the 364 retained rice accessions based on pericarp colouration. X‐axis = pericarp colour. Y‐axis = the number of accessions present in each colour group.

Figure S3 Boxplots of geometric, colour and nutritionally‐related traits assessed in the 364 retained rice accessions. The box plots show quantitative trait variation between the rice accessions grouped by pericarp colour. The pericarp colour was visually classified.

Figure S4 Histograms show the distribution of geometric, colour and nutritionally‐related traits in the 364 retained rice accessions.

Figure S5 Pearson correlations between the geometric, colour and nutritionally‐related traits assessed in the 364 retained rice accessions. The intensity of the blue (positive correlation) and red (negative correlation) colouration defines the value of the correlation coefficient. Insignificant correlations are not included in the figure.

Figure S6 The distribution of the ratio of Ho/He. Outlier SNPs were removed.

Figure S7 Proportion of heterozygous calls versus allele frequency (a) before filtering and (b) after filtering. Each dot represents a SNP.

Figure S8 Visual representation of the distribution and density of SNP markers across the 12 rice chromosomes.

Figure S9 Linkage disequilibrium (LD) decay. (a) LD decay plot for indica accessions, (b) LD decay plot for japonica accessions, (c) LD decay using the whole dataset.

Figure S10 Principal component analysis (PCA) of the 364 retained rice accessions. The additive relationship matrix was calculated in rrBLUP using the R package rrBLUP (Endelman, 2011) and used as input to the PCA. Colour‐coding is based on the region of origin. No structurization based on geographic origin was observed.

Figure S11 Genome‐wide association analysis of the geometric, colour and nutritionally‐related traits in the 364 retained rice accessions. Manhattan plots for each trait are shown for (A) Efficient Mixed‐Model Association eXpedited Model (EMMAX) and (B) multiple‐locus linear mixed model (MMLM). Bonferroni adjusted P value of −log10(P) = 6.47 was used as a threshold p‐value (red line). A nominal threshold of p = 1 × 10‐5 was also applied (blue line). (C) Possible genomic inflation was visualized using quantile‐quantile plots using the observed against the expected –log10 (P values). The X‐axis show the SNPs along each chromosome; Y axis is the—log10(P‐value) for the association.

Figure S12 Association network summary of candidate genes linked with geometric, colour‐related and nutritional traits. Gene‐targeted association analysis identified candidate genes associated with multiple nutritional traits, and colour‐related traits.

Figure S13 Haplotype analysis of significant SNPs identified in the Rc/bHLH gene. Phenotypic variation seen within the 364 retained rice accessions grouped by the four haplotypes identified within the Rc gene.

Figure S14 Haplotype analysis of significant SNPs identified in the IPT5 gene. Phenotypic variation seen within the 364 retained rice accessions grouped by the four haplotypes identified within the IPT5 gene.

Table S1: The 376 rice accessions from the International Rice Research Institute genebank that were genotyped by sequencing
Table S2: Videometer parameters in 364 rice accessions retained for further analyses
Table S3: Nutritional parameters measured in 364 retained rice accessions
Table S4: Descriptive statistics of rice seed geometric, colour and nutritional traits
Table S5: SNP marker and linkage disequilibrium statisitics per rice chromosome
Table S6: The significant SNP markers detected using EMMAX after applying the nominal threshold of p ≤ 1 × 10^‐5^
Table S7: The significant SNP markers detected using MLMM after applying the nominal threshold of p ≤ 1 × 10^‐5^
Table S8: The significant SNP markers detected using EMMAX after applying the Bonferroni correction −log10(P) = 6.1
Table S9: The significant SNP markers detected using MLMM after applying the Bonferroni correction −log10(P) = 6.1
Table S10: The significant SNP markers detected using EMMAX after applying the Bonferroni correction −log10(P) = 6.1 using the phenolic compound binary data
Table S11: A total of 169 multi‐traits loci were identified for significant SNPs identified using EMMAX after applying the nominal threshold of ≤ 1 × 10^‐5^
Table S12: A total of 79 multi‐traits loci were identified for significant SNPs identified using EMMAX after applying the Bonferroni correction
Table S13: A total of 10 multi‐traits loci were identified for significant SNPs identified using MLMM after applying the nominal threshold of ≤ 1 × 10^‐5^
Table S14: A total of 6 multi‐traits loci were identified for significant SNPs identified using MLMM after applying the Bonferroni correction
Table S15 Targeted gene association analysis for candidate genes linked with geometric, colour‐related and nutritional traits, filtered at beta value less than −0.3 or greater than 0.3.

## Data Availability

All data are available in the manuscript and the supplementary materials section online.

## References

[tpg220360-bib-0001] Alexander, D. H. , & Lange, K. (2011). Enhancements to the ADMIXTURE algorithm for individual ancestry estimation. BMC Bioinformatics, 12, 246. 10.1186/1471-2105-12-246 21682921 PMC3146885

[tpg220360-bib-0002] Armstrong, R. N. (1997). Structure, catalytic mechanism, and evolution of the glutathione transferases. Chemical Research in Toxicology, 10, 2–18. 10.1021/tx960072x 9074797

[tpg220360-bib-0003] Ashraf, H. , Murtaza, I. , Nazir, N. , Wani, A. B. , Naqash, S. , & Husaini, A. M. (2017). Nutritional profiling of pigmented and scented rice genotypes of Kashmir Himalayas. Journal of Pharmacognosy and Phytochemistry, 6, 910–916.

[tpg220360-bib-0004] Berni, R. , Cantini, C. , Romi, M. , Hausman, J.‐F. , Guerriero, G. , & Cai, G. (2018). Agrobiotechnology goes wild: Ancient local varieties as sources of bioactives. International Journal of Molecular Sciences, 19, 2248. 10.3390/ijms19082248 30071603 PMC6121869

[tpg220360-bib-0005] Bioversity International, IRRI, and WARDA . (2007). Descriptors for wild and cultivated Rice (Oryza spp.). Bioversity International, Rome, Italy; International Rice Research Institute, Los Baños, Philippines; WARDA, Africa Rice Center, Cotonou, Benin. 10.4324/9780203403556_Rice

[tpg220360-bib-0006] Bradbury, P. J. , Zhang, Z. , Kroon, D. E. , Casstevens, T. M. , Ramdoss, Y. , & Buckler, E. S. (2007). TASSEL: Software for association mapping of complex traits in diverse samples. Bioinformatics, 23, 2633–2635. 10.1093/bioinformatics/btm308 17586829

[tpg220360-bib-0007] Brooks, S. A. , Yan, W. , Jackson, A. K. , & Deren, C. W. (2008). A natural mutation in rc reverts white‐rice‐pericarp to red and results in a new, dominant, wild‐type allele: Rc‐g. Theoretical and Applied Genetics, 117, 575–580. 10.1007/s00122-008-0801-8 18516586

[tpg220360-bib-0008] Brotman, Y. , Llorente‐Wiegand, C. , Oyong, G. , Badoni, S. , Misra, G. , Anacleto, R. , Parween, S. , Pasion, E. , Tiozon, R. N. , Anonuevo, J. J. , Deguzman, M. K. , Alseekh, S. , Mbanjo, E. G. N. , Boyd, L. A. , Fernie, A. R. , & Sreenivasulu, N. (2021). The genetics underlying metabolic signatures in a brown rice diversity panel and their vital role in human nutrition. Plant Journal, 106, 507–525. 10.1111/tpj.15182 33529453

[tpg220360-bib-0009] Chang, C. C. , Chow, C. C. , Tellier, L. C. , Vattikuti, S. , Purcell, S. M. , & Lee, J. J. (2015). Second‐generation PLINK: Rising to the challenge of larger and richer datasets. Gigascience, 4, 7. 10.1186/s13742-015-0047-8 25722852 PMC4342193

[tpg220360-bib-0010] Cummins, I. , Wortley, D. J. , Sabbadin, F. , He, Z. , Coxon, C. R. , Straker, H. E. , Sellars, J. D. , Knight, K. , Edwards, L. , Hughes, D. , Kaundun, S. S. , Hutchings, S.‐J. , Steel, P. G. , & Edwards, R. (2013). Key role for a glutathione transferase in multiple‐herbicide resistance in grass weeds. Proceedings of the National Academy of Sciences USA, 110, 5812–5817. 10.1073/pnas.1221179110 PMC3625300 23530204

[tpg220360-bib-0011] Dixon, D. P. , Skipsey, M. , & Edwards, R. (2010). Roles for glutathione transferases in plant secondary metabolism. Phytochemistry, 71, 338–350. 10.1016/j.phytochem.2009.12.012 20079507

[tpg220360-bib-0012] Dong, Y. , Xu, J. , Xiao, K. E. , Zhang, Y. , Zhang, J. , Luo, L. , & Matsuo, M. (2008). Genomic regions associated with the degree of red coloration in pericarp of rice (*Oryza sativa* L.). Journal of Cereal Science, 48, 556–560. 10.1016/j.jcs.2007.11.011

[tpg220360-bib-0013] Dr Riar Cs, B. F. (2015). Health benefits of traditional rice varieties of temperate regions. Medicinal and Aromatic Plants, 4, 3–5. 10.4172/2167-0412.1000198

[tpg220360-bib-0014] Elmasry, G. , Mandour, N. , Al‐Rejaie, S. , Belin, E. , & Rousseau, D. (2019). Recent applications of multispectral imaging in seed phenotyping and quality monitoring—An overview. Sensors (Switzerland), 19, 1090. 10.3390/s19051090 PMC6427362 30836613

[tpg220360-bib-0015] Elshire, R. J. , Glaubitz, J. C. , Sun, Q. I. , Poland, J. A. , Kawamoto, K. , Buckler, E. S. , & Mitchell, S. E. (2011). A robust, simple genotyping‐by‐sequencing (GBS) approach for high diversity species. PLoS One, 6, e19379. 10.1371/journal.pone.0019379 21573248 PMC3087801

[tpg220360-bib-0016] Endelman, J. B. (2011). Ridge regression and other kernels for genomic selection with R Package rrBLUP. Plant Genome, 4, 250–255. 10.3835/plantgenome2011.08.0024

[tpg220360-bib-0017] Frank, T. , Reichardt, B. , Shu, Q. , & Engel, K.‐H. (2012). Metabolite profiling of colored rice (*Oryza sativa* L.) grains. Journal of Cereal Science, 55, 112–119. 10.1016/j.jcs.2011.09.009

[tpg220360-bib-0018] Furukawa, T. , Maekawa, M. , Oki, T. , Suda, I. , Iida, S. , Shimada, H. , Takamure, I. , & Kadowaki, K.‐I. (2006). The Rc and Rd genes are involved in proanthocyanidin synthesis in rice pericarp. Plant Journal, 49, 91–102. 10.1111/j.1365-313X.2006.02958.x 17163879

[tpg220360-bib-0019] Grosjean, P. , Frederic, I. , & Etienne, M. (2018). Package “pastecs”: Package for Analysis of Space‐Time Ecological Series. https://cran.r‐project.org/web/packages/pastecs/pastecs.pdf

[tpg220360-bib-0020] Gross, B. L. , Steffen, F. T. , & Olsen, K. M. (2010). The molecular basis of white pericarps in African domesticated rice: Novel mutations at the Rc gene. Journal of Evolutionary Biology, 23, 2747–2753. 10.1109/TMI.2012.2196707.Separate 21121088 PMC3058869

[tpg220360-bib-0021] Gunaratne, A. , Wu, K. , Li, D. , Bentota, A. , Corke, H. , & Cai, Y. I.‐Z. (2013). Antioxidant activity and nutritional quality of traditional red‐grained rice varieties containing proanthocyanidins. Food Chemistry, 138, 1153–1161. 10.1016/j.foodchem.2012.11.129 23411226

[tpg220360-bib-0022] Harrell, Jr, F. E. , Dupont, H. , & Al, E. (2020). Package “Hmisc.” https://cran.r‐project.org/web/packages/Hmisc/Hmisc.pdf

[tpg220360-bib-0023] Haynes, W. (2013). Bonferroni correction. In W. Dubitzky , O. Wolkenhauer , K. H. Cho , & H. Yokota (Eds.), Encyclopedia of systems biology (p. 154). Springer. 10.1007/978-1-4419-9863-7_1213

[tpg220360-bib-0024] He, J. , Zhao, X. , Laroche, A. , Lu, Z.‐X. , Liu, H. , & Li, Z. (2014). Genotyping‐by‐sequencing (GBS), An ultimate marker‐assisted selection (MAS) tool to accelerate plant breeding. Frontiers in Plant Science, 5, 484. 10.3389/fpls.2014.00484 25324846 PMC4179701

[tpg220360-bib-0025] Hu, J. , Anderson, B. , & Wessler, S. R. (1996). Isolation and characterization of rice R genes: Evidence for distinct evolutionary paths in rice and maize. Genetics, 142, 1021–1031. 10.1093/genetics/142.3.1021 8849907 PMC1207001

[tpg220360-bib-0026] Hua, Z. , Zou, C. , Shiu, S.‐H. , & Vierstra, R. D. (2011). Phylogenetic comparison of F‐Box (FBX) gene superfamily within the plant kingdom reveals divergent evolutionary histories indicative of genomic drift. PLoS One, 6(1), e16219. 10.1371/journal.pone.0016219 21297981 PMC3030570

[tpg220360-bib-0027] Jiang, H.‐W. , Liu, M.‐J. , Chen, I.‐C. , Huang, C.‐H. , Chao, L. I.‐Y. A. , & Hsieh, H.‐L. (2010). A glutathione S‐transferase regulated by light and hormones participates in the modulation of *Arabidopsis* seedling development. Plant Physiology, 154, 1646–1658. 10.1104/pp.110.159152 20935176 PMC2996023

[tpg220360-bib-0028] Johnson, M. P. , Davison, P. A. , Ruban, A. V. , & Horton, P. (2008). The xanthophyll cycle pool size controls the kinetics of non‐photochemical quenching in *Arabidopsis thaliana* . FEBS Letters, 582, 262–266. 10.1016/j.febslet.2007.12.016 18083127

[tpg220360-bib-0029] Kamvar, Z. N. , Tabima, J. F. , & Grünwald, N. J. (2014). Poppr: An R package for genetic analysis of populations with clonal, partially clonal, and /or sexual reproduction. PeerJ, 2, e281. 10.7717/peerj.281 24688859 PMC3961149

[tpg220360-bib-0091] Kang, H. M. , Sul, J. H. , Service, S. K. , Zaitlen, N. A. , Kong, S. Y. , Freimer, N. B. , Sabatti, C. , & Eskin, E. (2010). Variance component model to account for sample structure in genome–wide association studies. Nature Genetics, 42(4), 348–354. 10.1038/ng.548 20208533 PMC3092069

[tpg220360-bib-0030] Katsumoto, Y. , Fukuchi‐Mizutani, M. , Fukui, Y. , Brugliera, F. , Holton, T. A. , Karan, M. , Nakamura, N. , Yonekura‐Sakakibara, K. , Togami, J. , Pigeaire, A. , Tao, G.‐Q. , Nehra, N. S. , Lu, C.‐Y. , Dyson, B. K. , Tsuda, S. , Ashikari, T. , Kusumi, T. , Mason, J. G. , & Tanaka, Y. (2017). Engineering of the rose flavonoid biosynthetic pathway successfully generated blue‐hued flowers accumulating delphinidin. Plant & Cell Physiology, 48(11), 1589–1600. 10.1093/pcp/pcm131 17925311

[tpg220360-bib-0032] Keenan, K. , Mcginnity, P. , Cross, T. F. , Crozier, W. W. , & Prodöhl, P. A. (2013). diveRsity: An R package for the estimation and exploration of population genetics parameters and their associated errors. Methods in Ecology and Evolution, 4, 782–788. 10.1111/2041-210X.12067

[tpg220360-bib-0033] Lê, S. , Josse, J. , & Husson, F. (2008). FactoMineR: An R package for multivariate analysis. Journal of Statistical Software, 25, 1–18. 10.18637/jss.v025.i01

[tpg220360-bib-0034] Ludwig, D. S. , Hu, F. B. , Tappy, L. , & Brand‐Miller, J. (2018). Dietary carbohydrates: Role of quality and quantity in chronic disease. BMJ, 361, k2340. 10.1136/bmj.k2340 29898880 PMC5996878

[tpg220360-bib-0035] Ma, Y.‐J. , Duan, H.‐R. , Zhang, F. , Li, Y. , Yang, H.‐S. , Tian, F.‐P. , Zhou, X.‐H. , Wang, C.‐M. , & Ma, R. (2018). Transcriptomic analysis of *Lycium ruthenicum* Murr. during fruit ripening provides insight into structural and regulatory genes in the anthocyanin biosynthetic pathway. PLoS One, 13, e0208627. 10.1371/journal.pone.0208627 30532153 PMC6285980

[tpg220360-bib-0036] Maeda, H. , Yamaguchi, T. , Omoteno, M. , Takarada, T. , Fujita, K. , Murata, K. , Iyama, Y. , Kojima, Y. , Morikawa, M. , Ozaki, H. , Mukaino, N. , Kidani, Y. , & Ebitani, T. (2014). Genetic dissection of black grain rice by the development of a near isogenic line. Breeding Science, 64, 134–141. 10.1270/jsbbs.64.134 24987299 PMC4065320

[tpg220360-bib-0037] Matsuda, F. , Okazaki, Y. , Oikawa, A. , Kusano, M. , Nakabayashi, R. , Kikuchi, J. , Yonemaru, J.‐I. , Ebana, K. , Yano, M. , & Saito, K. (2012). Dissection of genotype–phenotype associations in rice grains using metabolome quantitative trait loci analysis. Plant Journal, 70, 624–636. 10.1111/j.1365-313X.2012.04903.x 22229385

[tpg220360-bib-0038] Mbanjo, E. G. N. , Jones, H. , Caguiat, X. G. I. , Carandang, S. , Ignacio, J. C. , Ferrer, M. C. , Boyd, L. A. , & Kretzschmar, T. (2019). Exploring the genetic diversity within traditional Philippine pigmented rice. Rice, 12, 27. 10.1186/s12284-019-0281-2 31041567 PMC6491523

[tpg220360-bib-0039] Mbanjo, E. G. N. , Kretzschmar, T. , Jones, H. , Ereful, N. , Blanchard, C. , Boyd, L. A. , & Sreenivasulu, N. (2020). The genetic basis and nutritional benefits of pigmented rice grain. Frontiers in Genetics, 11, 229. 10.3389/fgene.2020.00229 32231689 PMC7083195

[tpg220360-bib-0040] Melini, V. , & Acquistucci, R. (2017). Health‐promoting compounds in pigmented Thai and wild rice. Foods, 6, E9. 10.3390/foods6010009 PMC5296678 28231088

[tpg220360-bib-0041] Mgonja, E. M. , Park, C. H. , Kang, H. , Balimponya, E. G. , Opiyo, S. , Bellizzi, M. , Mutiga, S. K. , Rotich, F. , Ganeshan, V. D. , Mabagala, R. , Sneller, C. , Correll, J. , Zhou, B. , Talbot, N. J. , Mitchell, T. K. , & Wang, G.‐L. (2017). Genotyping‐by‐sequencing‐based genetic analysis of African rice cultivars and association mapping of blast resistance genes against *Magnaporthe oryzae* populations in Africa. Phytopathology, 107, 1039–1046. 10.1094/PHYTO-12-16-0421-R 28719243

[tpg220360-bib-0042] Misra, G. , Badoni, S. , Parween, S. , Singh, R. K. , Leung, H. , Ladejobi, O. , Mott, R. , & Sreenivasulu, N. (2020). Genome‐wide association coupled gene to gene interaction studies unveil novel epistatic targets among major effect loci impacting rice grain chalkiness. Plant Biotechnology Journal, 19, 910–925. 10.1111/pbi.13516 33220119 PMC8131057

[tpg220360-bib-0043] Murray, K. D. , & Borevitz, J. O. (2018). Axe: Rapid, competitive sequence read demultiplexing using a trie. Bioinformatics, 34, 3924–3925. 10.1093/bioinformatics/bty432 29868827

[tpg220360-bib-0044] Nagashima, Y. , Tsugawa, S. , Mochizuki, A. , Sasaki, T. , Fukuda, H. , & Oda, Y. (2018). A Rho‐based reaction‐diffusion system governs cell wall patterning in metaxylem vessels. Scientific Reports, 8(2018), 11542. 10.1038/s41598-018-29543-y 30069009 PMC6070580

[tpg220360-bib-0045] Nagy, F. , & Schäfer, E. (2002). Phytochromes control photomorphogenesis by differentially regulated, interacting signalling pathways in higher plants. Annual Review of Plant Biology, 53, 329–355. 10.1146/annurev.arplant.53.100301.135302 12221979

[tpg220360-bib-0046] Nguyen, H. N. , Lai, N. , Kisiala, A. B. , & Emery, R. J. N. (2021). Isopentenyltransferases as master regulators of crop performance: Their function, manipulation, and genetic potential for stress adaptation and yield improvement. Plant Biotechnology Journal, 19(7), 1297–1313. 10.1111/pbi.13603 33934489 PMC8313133

[tpg220360-bib-0047] Oikawa, T. , Maeda, H. , Oguchi, T. , Yamaguchi, T. , Tanabe, N. , Ebana, K. , Yano, M. , Ebitani, T. , & Izawa, T. (2015). The birth of a black rice gene and its local spread by introgression. Plant Cell, 27, 2401–2414. 10.1105/tpc.15.00310 26362607 PMC4815089

[tpg220360-bib-0048] Oko, A. O. , Ubi, B. E. , Efisue, A. A. , & Dambaba, N. (2012). Comparative analysis of the chemical nutrient composition of selected local and newly introduced rice varieties grown in ebonyi state of Nigeria. International Journal of Agriculture, 2, 16–23. 10.5923/j.ijaf.20120202.04

[tpg220360-bib-0049] OMS . (2016). Rapport mondial sur le diabète. Organisation mondiale de la Santé.

[tpg220360-bib-0050] Paradis, E. , Claude, J. , & Strimmer, K. (2004). APE: Analyses of phylogenetics and evolution in R language. Bioinformatics, 20, 289–290. 10.1093/bioinformatics/btg412 14734327

[tpg220360-bib-0092] Purcell, S. , Neale, B. , Todd–Brown, K. , Thomas, L. , Ferreira, M. A. , Bender, D. , Maller, J. , Sklar, P. , de Bakker, P. I. , Daly, M. J. , & Sham, P. C. (2007). PLINK: A tool set for whole‐genome association and population‐based linkage analyses. American Journal of Human Genetics, 81(3), 559–575. 10.1086/519795 17701901 PMC1950838

[tpg220360-bib-0051] Rachasima, L. N. , Sukkasem, R. , Pongjaroenkit, S. , Sangtong, V. , Chowpongpang, S. , & Sakulsingharoj, C. (2017). Expression analysis and nucleotide variation of OsC1 gene associated with anthocyanin pigmentation in Thai rice cultivars. Genomics Genet, 10, 46–53.

[tpg220360-bib-0052] Rathna Priya, T. S. , Eliazer Nelson, A. R. L. , Ravichandran, K. , & Antony, U. (2019). Nutritional and functional properties of pigmented rice varieties of South India: A review. Journal of Ethnic Foods, 6, 11. 10.1186/s42779-019-0017-3

[tpg220360-bib-0053] Rausenberger, J. , Hussong, A. , Kircher, S. , Kirchenbauer, D. , Timmer, J. , Nagy, F. , Schäfer, E. , & Fleck, C. (2010). An integrative model for phytochrome B mediated photomorphogenesis: From protein dynamics to physiology. PLoS One, 5(2010), e10721. 10.1371/journal.pone.0010721 20502669 PMC2873432

[tpg220360-bib-0055] RStudio Team . (2016). RStudio: Integrated Development for R. RStudio, Inc . http://www.rstudio.com

[tpg220360-bib-0056] Saitoh, K. , Onishi, K. , Mikami, I. , Thidar, K. , & Sano, Y. (2004). Allelic diversification at the C (OsC1) locus of wild and cultivated rice: Nucleotide changes associated with phenotypes. Genetics, 168, 997–1007. 10.1534/genetics.103.018390 15514070 PMC1448844

[tpg220360-bib-0057] Sakulsingharoj, C. , Inta, P. , Sukkasem, R. , Pongjaroenkit, S. , Chowpongpang, S. , & Sangtong, V. (2016). Cloning and characterization of *OSB1* gene controlling anthocyanin biosynthesis from Thai black rice. Genomics Genet, 9, 7–18. 10.14456/gag.2016.2

[tpg220360-bib-0058] Samyor, D. , Das, A. B. , & Deka, S. C. (2017). Pigmented rice a potential source of bioactive compounds: A review. International Journal of Food Science and Technology, 52, 1073–1081. 10.1111/ijfs.13378

[tpg220360-bib-0059] Saneei, P. , Larijani, B. , & Esmaillzadeh, A. (2016). Review article rice consumption, incidence of chronic diseases and risk of mortality: Meta‐analysis of cohort studies. Public Health Nutrition, 20, 233–244. 10.1017/S1368980016002172 27577106 PMC10261662

[tpg220360-bib-0060] Sarma, R. , Kumara Swamy, H. V. V. , & Shashidhar, H. E. (2018). Dealing with zinc and iron deficiency in rice: Combine strategies to fight hidden hunger in developing countries. International Journal of Current Microbiology and Applied Science, 7, 1887–1895. 10.20546/ijcmas.2018.703.224

[tpg220360-bib-0061] Segura, V. , Vilhjálmsson, B. J. , Platt, A. , Korte, A. , Seren, Ü. , Long, Q. , & Nordborg, M. (2012). An efficient multi‐locus mixed‐model approach for genome‐wide association studies in structured populations. Nature Genetics, 44, 825–830. 10.1038/ng.2314 22706313 PMC3386481

[tpg220360-bib-0062] Shannon, P. , Markiel, A. , Ozier, O. , Baliga, N. S. , Wang, J. T. , Ramage, D. , Amin, N. , Schwikowski, B. , & Ideker, T. (2003). Cytoscape: A software environment for integrated models of biomolecular interaction networks. Genome Research, 13(11), 2498–504. 10.1101/gr.1239303 14597658 PMC403769

[tpg220360-bib-0063] Shao, Y. , Hu, Z. , Yu, Y. , Mou, R. , Zhu, Z. , & Beta, T. (2018). Phenolic acids, anthocyanis, proanthocyanidins, antioxidant activity, minerals and their correlations in non‐pigmented, red, and black rice. Food Chemistry, 239, 733–741. 10.1016/j.foodchem.2017.07.009 28873629

[tpg220360-bib-0064] Shao, Y. , Jin, L. , Zhang, G. , Lu, Y. , Shen, Y. , & Bao, J. (2011). Association mapping of grain color, phenolic content, flavonoid content and antioxidant capacity in dehulled rice. Tag. Theoretical and Applied Genetics Theoretische Und Angewandte Genetik, 122, 1005–1016. 10.1007/s00122-010-1505-4 21161500

[tpg220360-bib-0065] Sharma, A. , Patni, B. , Shankhdhar, D. , & Shankhdhar, S. C. (2013). Zinc—An indispensable micronutrient. Physiology and Molecular Biology of Plants, 19, 11–20. 10.1007/s12298-012-0139-1 24381434 PMC3550680

[tpg220360-bib-0066] Singh, N. , Singh, B. , Rai, V. , Sidhu, S. , Singh, A. K. , & Singh, N. K. (2017). Evolutionary insights based on SNP haplotypes of red pericarp, grain size and starch synthase genes in wild and cultivated rice. Frontiers in Plant Science, 8, 972. 10.3389/fpls.2017.00972 28649256 PMC5465369

[tpg220360-bib-0067] Sompong, R. , Siebenhandl‐Ehn, S. , Linsberger‐Martin, G. , & Berghofer, E. (2011). Physicochemical and antioxidative properties of red and black rice varieties from Thailand, China and Sri Lanka. Food Chemistry, 124, 132–140. 10.1016/j.foodchem.2010.05.115

[tpg220360-bib-0068] Song, X.‐J. , Huang, W. , Shi, M. , Zhu, M.‐Z. , & Lin, H.‐X. (2007). A QTL for rice grain width and weight encodes a previously unknown RING‐type E3 ubiquitin ligase. Nature Genetics, 39, 623–630. 10.1038/ng2014 17417637

[tpg220360-bib-0069] Sun, D. , Cen, H. , Weng, H. , Wan, L. , Abdalla, A. , El‐Manawy, A. I. , Zhu, Y. , Zhao, N. , Fu, H. , Tang, J. , Li, X. , Zheng, H. , Shu, Q. , Liu, F. , & He, Y. (2019). Using hyperspectral analysis as a potential high throughput phenotyping tool in GWAS for protein content of rice quality. Plant Methods, 15, 54. 10.1186/s13007-019-0432-x 31139243 PMC6532189

[tpg220360-bib-0070] Sun, X. , Zhang, Z. , Chen, C. , Wu, W. , Ren, N. , Jiang, C. , Yu, J. , Zhao, Y. , Zheng, X. , Yang, Q. , Zhang, H. , Li, J. , & Li, Z. (2018). The C–S–A gene system regulates hull pigmentation and reveals evolution of anthocyanin biosynthesis pathway in rice. Journal of Experimental Botany, 69, 1485–1498. 10.1093/jxb/ery001 29361187 PMC5888925

[tpg220360-bib-0071] Sweeney, M. T. , Thomson, M. J. , Cho, Y. G. , Park, Y. J. , Williamson, S. H. , Bustamante, C. D. , & Mccouch, S. R. (2007). Global dissemination of a single mutation conferring white pericarp in rice. Plos Genetics, 3, e133. 10.1371/journal.pgen.0030133 17696613 PMC1941752

[tpg220360-bib-0072] Sweeney, M. T. , Thomson, M. J. , Pfeil, B. E. , & Mccouch, S. (2006). Caught red‐handed: Rc Encodes a basic helix‐loop‐helix protein conditioning red pericarp in rice. Plant Cell, 18, 283–294. 10.1105/tpc.105.038430.1 16399804 PMC1356539

[tpg220360-bib-0073] Tan, Y. F. , Sun, M. , Xing, Y. Z. , Hua, J. P. , Sun, X. L. , Zhang, Q. F. , & Corke, H. (2001). Mapping quantitative trait loci for milling quality, protein content and color characteristics of rice using a recombinant inbred line population derived from an elite rice hybrid. Theoretical and Applied Genetics, 103, 1037–1045. 10.1007/s001220100665

[tpg220360-bib-0074] United Nations Department of Economic and Social Affairs, Population Division . (2022). World Population Prospects 2022: Summary of Results . https://www.un.org/development/desa/pd/content/World‐Population‐Prospects‐2022

[tpg220360-bib-0075] Valarmathi, R. , Raveendran, M. , Robin, S. , & Senthil, N. (2014). Unravelling the nutritional and therapeutic properties of ‘Kavuni’ a traditional rice variety of Tamil Nadu. Journal of Plant Biochemistry and Biotechnology, 24, 305–315. 10.1007/s13562-014-0274-6

[tpg220360-bib-0076] Vartapetian, A. , Tuzhikov, A. , Chichkova, N. , Taliansky, M. , & Wolpert, T. (2011). Agrobiotechnology goes wild: Ancient local varieties as sources of bioactives. International Journal of Molecular Sciences, 19, 2248. 10.3390/ijms19082248 PMC6121869 30071603

[tpg220360-bib-0077] Verma, D. K. , & Shukla, K. (2011). Nutritional value of rice and their importance. Indian Farmers Digging, 44, 21–35.

[tpg220360-bib-0078] Wang, K. , Li, M. , & Hakonarson, H. (2010). ANNOVAR: Functional annotation of genetic variants from high‐throughput sequencing data. Nucleic Acids Research, 38(16), e164. 10.1093/nar/gkq603 20601685 PMC2938201

[tpg220360-bib-0079] Weir, B. S. , & Cockerham, C. C. (1984). Estimating F‐statistics for the analysis of population structure. Evolution; Internation Journal of Organic Evolution, 38, 1358–1370. 10.1111/j.1558-5646.1984.tb05657.x 28563791

[tpg220360-bib-0080] Wickham, H. (2016). ggplot2: Elegant Graphics for Data Analysis. Springer‐Verlag New York. https://ggplot2.tidyverse.org

[tpg220360-bib-0081] Xu, T. Y. , Sun, J. , Chang, H. L. , Zheng, H. L. , Wang, J. G. , Liu, H. L. , Yang, L. M. , Zhao, H. W. , & Zou, D. T. (2017). QTL mapping for anthocyanin and proanthocyanidin content in red rice. Euphytica, 213, 243. 10.1007/s10681-017-2035-9

[tpg220360-bib-0082] Yang, X. , Xia, X. , Zeng, Y. , Nong, B. , Zhang, Z. , Wu, Y. , Xiong, F. , Zhang, Y. , Liang, H. , Deng, G. , & Li, D. (2018). Identification of candidate genes for gelatinization temperature, gel consistency and pericarp color by GWAS in rice based on SLAF‐sequencing. Plos Genetics, 13, e0196690. 10.1371/journal.pone.01 PMC5944943 29746484

[tpg220360-bib-0083] Yean, R. U.‐A. , Dilipkumar, M. , Rahman, S. , & Song, B.‐K. (2021). A two‐in‐one strategy: Target and nontarget site mechanisms both play important role in IMI‐resistant weedy rice. International Journal of Molecular Sciences, 22(3), 982. 10.3390/ijms22030982 33498150 PMC7863736

[tpg220360-bib-0084] Yu, G. , Smith, D. K. , Zhu, H. , Guan, Y. I. , & Lam, T. T.‐Y. (2017). GGTREE: An R package for visualization and annotation of phylogenetic trees with their covariates and other associated data. Methods in Ecology and Evolution, 8, 28–36. 10.1111/2041-210X.12628

[tpg220360-bib-0086] Zhang, C. , Tan, Y. , Essemine, J. , Xu, Q. , Fang, B. , Tan, Y. , Li, N. , Liu, H. , Qu, M. , & Wang, W. (2020). Identification of stress repressive zinc finger gene family and its expression analysis in rice under abiotic constraints, 29 January 2020, PREPRINT (Version 1). Research Square. 10.21203/rs.2.22108/v1

[tpg220360-bib-0088] Zhang, Y. , Xu, S. , Cheng, Y. , Peng, Z. , & Han, J. (2018). Transcriptome profiling of anthocyanin‐related genes reveals effects of light intensity on anthocyanin biosynthesis in red leaf lettuce. PeerJ, 6, e4607. 10.7717/peerj.4607 29666761 PMC5900932

[tpg220360-bib-0089] Zheng, K. , Pang, L. , Xue, X. , Gao, P. , Zhao, H. , Wang, Y. , & Han, S. (2022). Genome‐Wide Comprehensive survey of the subtilisin‐like proteases gene family associated with rice caryopsis development. Frontiers in Plant Science, 13, 943184. 10.3389/fpls.2022.943184 35795345 PMC9251471

