# Supplementary material for: Unravelling marker trait associations linking nutritional value with pigmentation in rice seed
Source: Plant Genome. 2023 Aug 17;16(4):e20360. doi: 10.1002/tpg2.20360 (PMC12806873; doi:10.1002/tpg2.20360)
Supplement: Supplementary file 1 [file TPG2-16-e20360-s006.pdf]

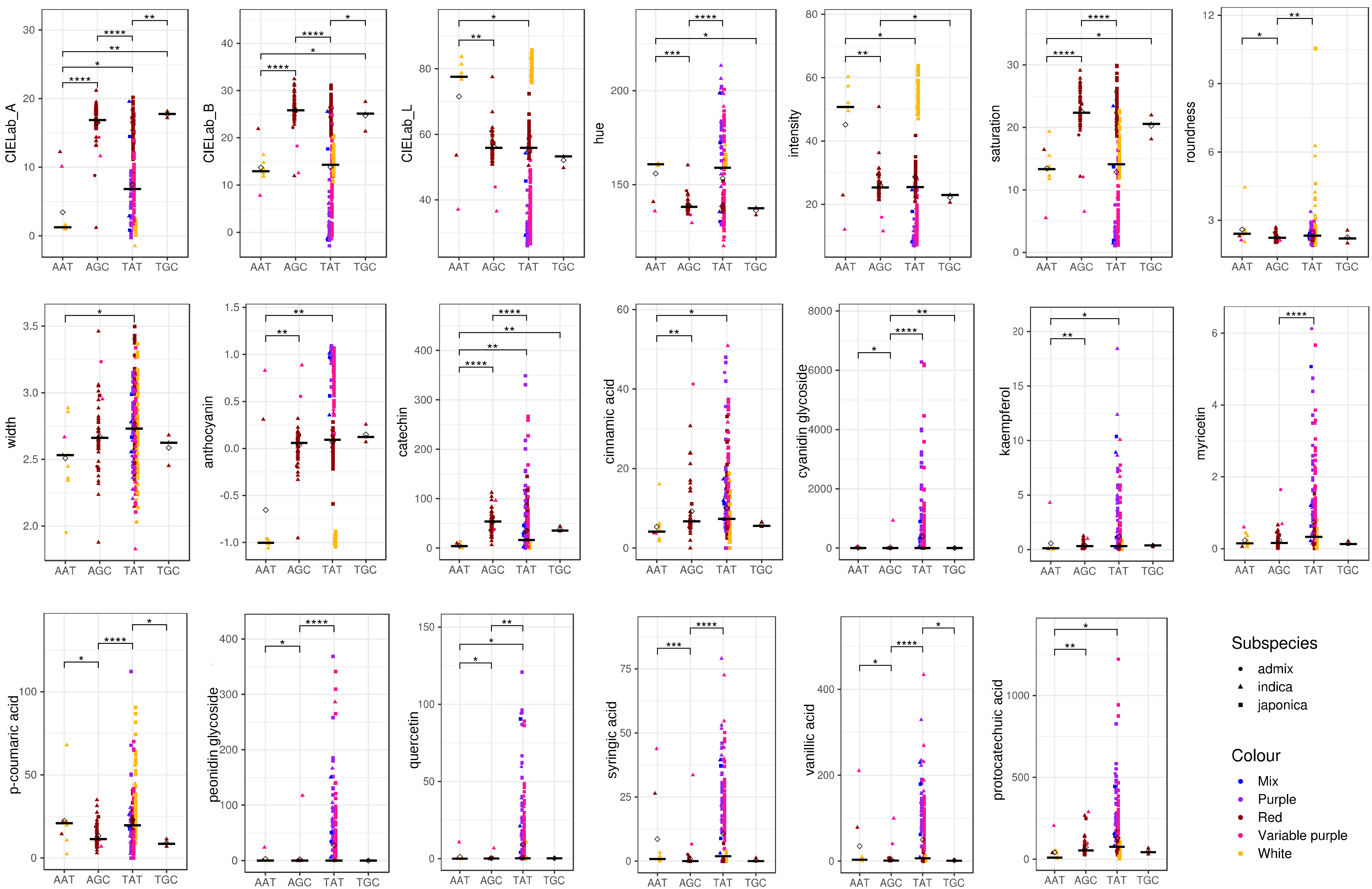

CIELab_A
CIELab_B
CIELab_L
hue
intensity
saturation
roundness
width
anthocyanin
catechin
cinnamic acid
cyanidin glycoside
kaempferol
myricetin
p-coumaric acid
peonidin glycoside
quercetin
syringic acid
vanillic acid
protocatechuic acid
AAT
AGC
TAT
TGC
Subspecies
admix
indica
japonica
Colour
Mix
Purple
Red
Variable purple
White
