# Supplementary material for: Unravelling marker trait associations linking nutritional value with pigmentation in rice seed
Source: Plant Genome. 2023 Aug 17;16(4):e20360. doi: 10.1002/tpg2.20360 (PMC12806873; doi:10.1002/tpg2.20360)
Supplement: Supplementary file 2 [file TPG2-16-e20360-s014.pdf]

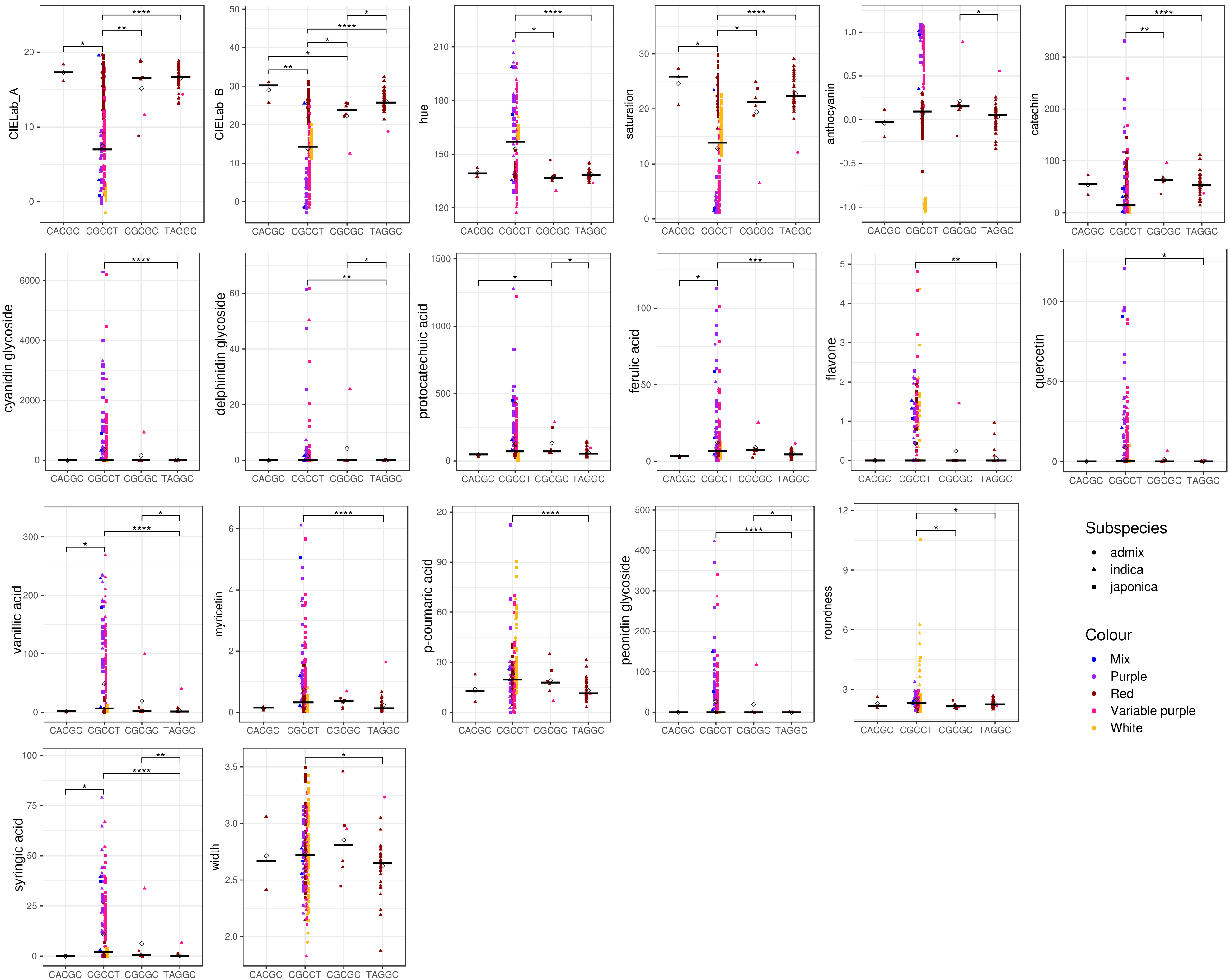
CIELab_A
CIELab_B
hue
saturation
anthocyanin
catechin
cyanidin glycoside
delphinidin glycoside
protocatechuic acid
ferulic acid
flavone
quercetin
vanillic acid
myricetin
p-coumaric acid
peonidin glycoside
roundness
syringic acid
width
CACGC CGCCT CGCGC TAGGC
Subspecies
admix
indica
japonica
Colour
Mix
Purple
Red
Variable purple
White
