# Supplementary material for: Unravelling marker trait associations linking nutritional value with pigmentation in rice seed
Source: Plant Genome. 2023 Aug 17;16(4):e20360. doi: 10.1002/tpg2.20360 (PMC12806873; doi:10.1002/tpg2.20360)
Supplement: Supplementary file 3 [file TPG2-16-e20360-s015.pdf]

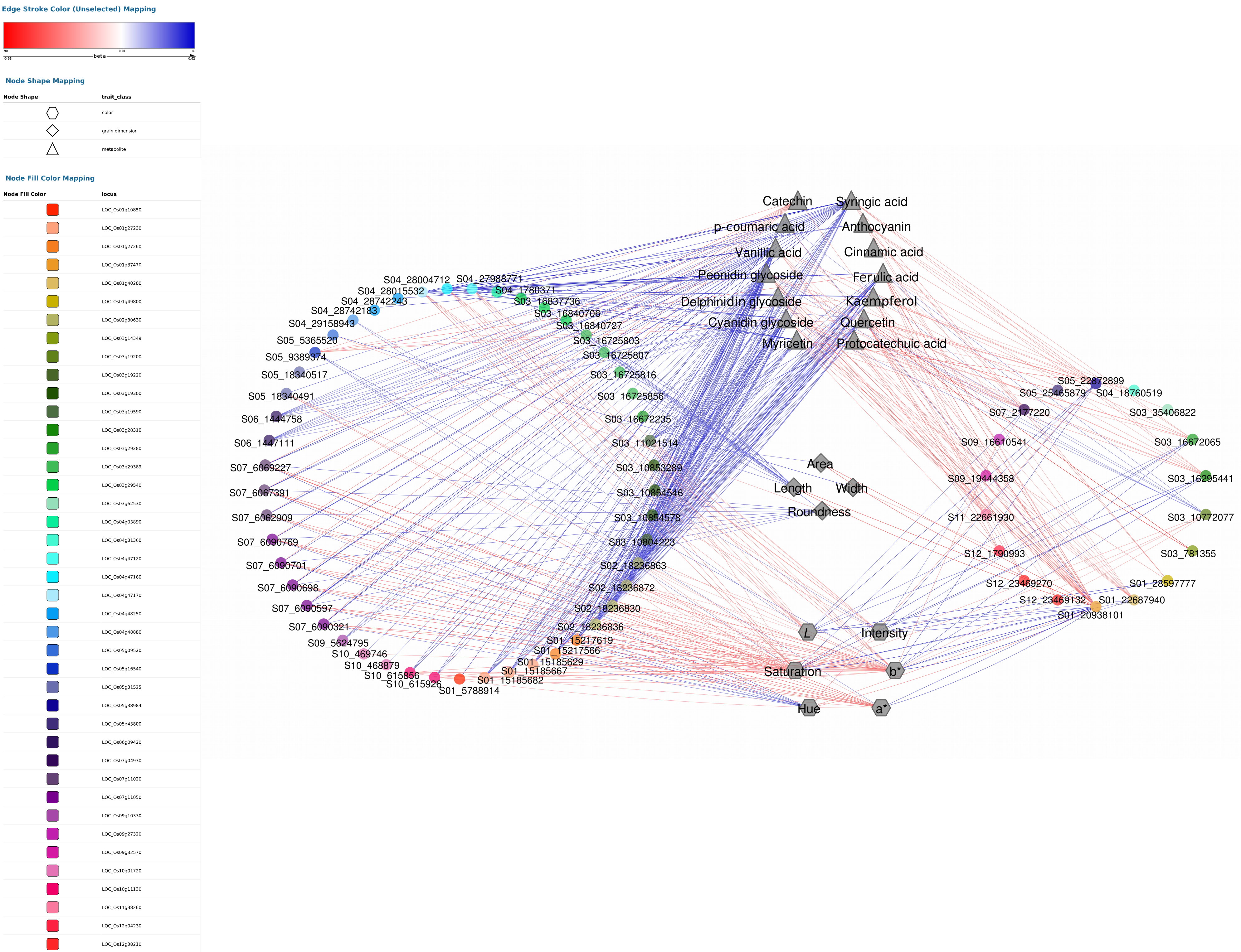

Edge Stroke Color (Unselected) Mapping
beta
-0.98
0.62
Node Shape Mapping
Node Shape
trait_class
color
grain dimension
metabolite
Node Fill Color Mapping
Node Fill Color
locus
LOC_Os01g10850
LOC_Os01g27230
LOC_Os01g27260
LOC_Os01g37470
LOC_Os01g40200
LOC_Os01g49800
LOC_Os02g30630
LOC_Os03g14349
LOC_Os03g19200
LOC_Os03g19220
LOC_Os03g19300
LOC_Os03g19590
LOC_Os03g28310
LOC_Os03g29280
LOC_Os03g29389
LOC_Os03g29540
LOC_Os03g62530
LOC_Os04g03890
LOC_Os04g31360
LOC_Os04g47120
LOC_Os04g47160
LOC_Os04g47170
LOC_Os04g48250
LOC_Os04g48880
LOC_Os05g09520
LOC_Os05g16540
LOC_Os05g31525
LOC_Os05g38984
LOC_Os05g43800
LOC_Os06g09420
LOC_Os07g04930
LOC_Os07g11020
LOC_Os07g11050
LOC_Os09g10330
LOC_Os09g27320
LOC_Os09g32570
LOC_Os10g01720
LOC_Os10g11130
LOC_Os11g38260
LOC_Os12g04230
LOC_Os12g38210
Catechin
Syringic acid
p-coumaric acid
Anthocyanin
Vanillic acid
Cinnamic acid
Peonidin glycoside
Ferulic acid
Delphinidin glycoside
Kaempferol
Cyanidin glycoside
Quercetin
Myricetin
Protocatechuic acid
Area
Length
Width
Roundness
L
Intensity
Saturation
b*
Hue
a*
S04_28004712
S04_27988771
S04_28015532
S04_1780371
S04_28742243
S03_16837736
S04_28742183
S03_16840706
S04_29158943
S03_16840727
S05_5365520
S03_16725803
S05_9389374
S03_16725807
S05_18340517
S03_16725816
S05_18340491
S03_16725856
S06_1444758
S03_16672235
S06_1447111
S03_11021514
S07_6069227
S03_10853289
S07_6067391
S03_10854546
S07_6062909
S03_10854578
S07_6090769
S03_10804223
S07_6090701
S02_18236863
S07_6090698
S02_18236872
S07_6090597
S02_18236830
S07_6090321
S02_18236836
S09_5624795
S01_15217619
S10_469746
S01_15217566
S10_468879
S01_15185629
S10_615856
S01_15185667
S10_615926
S01_15185682
S01_5788914
S05_22872899
S05_25465879
S04_18760519
S07_2177220
S03_35406822
S09_16610541
S03_16672065
S09_19444358
S03_16295441
S11_22661930
S03_10772077
S12_1790993
S03_781355
S12_23469270
S01_28597777
S12_23469132
S01_22687940
S01_20938101
